# Dual Structure Reinforces Interfacial Polarized MXene/PVDF-TrFE Piezoelectric Nanocomposite for Pressure Monitoring

**DOI:** 10.1007/s40820-025-01839-5

**Published:** 2025-07-04

**Authors:** Yong Ao, Long Jin, Shenglong Wang, Bolin Lan, Guo Tian, Tianpei Xu, Longchao Huang, Zihan Wang, Yue Sun, Tao Yang, Weili Deng, Fan Yang, Weiqing Yang

**Affiliations:** 1 Key Laboratory of Advanced Technologies of Materials, Ministry of Education, School of Materials Science and Engineering, Southwest Jiaotong University, Chengdu, 610031 People’s Republic of China; 2 Research Institute of Frontier Science, Southwest Jiaotong University, Chengdu, 610031 People’s Republic of China; 3 Shanghai Institute of Traumatology and Orthopaedics, Ruijin Hospital, Shanghai Jiao Tong University School of Medicine, Shanghai, 200025 People’s Republic of China

**Keywords:** Piezoelectric composite, MXene/PVDF-TrFE, Interfacial polarization, Structural engineering

## Abstract

The emerging interfacial polarization strategy exhibits applicative potential in piezoelectric enhancement. However, there is an ongoing effort to address the inherent limitations arising from charge bridging phenomena and stochastic interface disorder that plague the improvement of piezoelectric performance. Here, we report a dual structure reinforced MXene/PVDF-TrFE piezoelectric composite, whose piezoelectricity is enhanced under the coupling effect of interfacial polarization and structural design. Synergistically, molecular dynamics simulations, density functional theory calculations and experimental validation revealed the details of interfacial interactions, which promotes the net spontaneous polarization of PVDF-TrFE from the 0.56 to 31.41 Debye. The oriented MXene distribution and porous structure not only tripled the piezoelectric response but also achieved an eightfold increase in sensitivity within the low-pressure region, along with demonstrating cyclic stability exceeding 20,000 cycles. The properties reinforcement originating from dual structure is elucidated through the finite element simulation and experimental validation. Attributed to the excellent piezoelectric response and deep learning algorithm, the sensor can effectively recognize the signals of artery pulse and finger flexion. Finally, a 3 × 3 sensor array is fabricated to monitor the pressure distribution wirelessly. This study provides an innovative methodology for reinforcing interfacial polarized piezoelectric materials and insight into structural designs.

**Supplementary Information:**

The online version contains supplementary material available at 10.1007/s40820-025-01839-5.

## Highlights

- The underlying mechanism improving piezoelectricity via interfacial polarization is elucidated through combining the experimental results, molecular dynamics simulations and density functional theory calculations.
- The piezoelectric performance of the nanocomposite is improved based on the successful construction of dual-structure.
- The piezoelectric sensor and array are capable of identifying human physiological signals and monitoring the distribution of pressure.

**Supplementary Information:**

The online version contains supplementary material available at 10.1007/s40820-025-01839-5.

## Graphical abstract

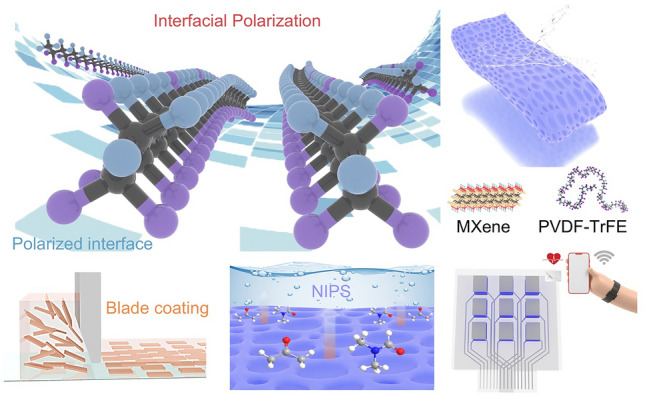

## Introduction

Piezoelectric materials play a critical role in sensing and actuating [1, 2]. Due to the flexibility, biocompatibility, and lightweight characteristics, polyvinylidene fluoride (PVDF) and its copolymers are widely utilized in wearable sensors, medical actuators and various flexible electronic devices [3–8]. Although PVDF exhibits reliable piezoelectricity and ferroelectricity, achieving these qualities requires intensive processing [9–13]. In comparison, poly(vinylidene fluoride-ran-trifluoroethylene) (PVDF-TrFE) features enhanced polarization capability, improved phase stability, and a higher piezoelectric coefficient, which can be attributed to the inclusion of TrFE units on the molecular chains [12, 14]. However, despite these advantages, substantial efforts are still required to further enhance the piezoelectric properties of PVDF-TrFE, as there remains a significant performance gap compared to ceramic piezoelectric materials. Numerous studies have been dedicated to enhancing the piezoelectric properties of PVDF-TrFE. Traditional approaches include incorporating fillers with high piezoelectricity or high dielectric constant into the matrix, as well as employing preparation methods that utilize in situ fields—such as electrospinning, hot pressing, and electrohydrodynamic printing—to improve material performance [15, 16]. In principle, polarization enhancement is the critical approach for boosting piezoelectric properties [17–20]. Up to now, tremendous efforts have been focused on structural design at the molecular scale, including chemical modification [20, 21], component regulation [14], and doping with functional fillers [22, 23].

The method of templating polarization has been investigated to induce interfacial polarization via the interactions between nanofillers and PVDF-TrFE at the interface [24–27]. It turns out to be a prospective pathway to realize piezoelectric performance enhancement by interfacial polarization, emphasizing the crucial role of a well-defined polarized interface in material design [28–31]. MXene, a burgeoning two-dimensional material, features a high aspect ratio and significant specific surface area [32–34]. More importantly, MXene possesses abundant terminal functional groups that provide a polar surface, making it an ideal candidate for creating a large well-polarized interface within the matrix to boost piezoelectric performance [35]. However, the random distribution of the polarized interface in the composite prepared by conventional methods can impair the full enhancement of piezoelectricity. In addition, given the conductive nature of MXene, a random distribution would risk unintended bridging effects which constrains the content of addition, potentially undermining the piezoelectric performance. The mutual constraints between interfacial polarization and bridging effects induced by MXene significantly hinder the enhancement of piezoelectric properties. To date, few innovative approaches have successfully reconciled these interdependent trade-offs through tailored structural designs, leaving a critical gap in overcoming such competing requirements. Moreover, implementing advanced structural engineering approaches shows promising potential for amplifying the piezoelectric response of nanocomposites, which could broaden their application scope in emerging technologies.

In this work, we constructed a dual structure in the piezoelectric PVDF-TrFE /MXene composite by integrating Ti_3_C_2_T_x_ MXene nanosheets into PVDF-TrFE matrix through a combination of blade coating and non-solvent induce phase separation (NIPS). This process results in a composite with oriented interface distribution and a porous microstructure, which is critical for enhancing piezoelectric properties. To elucidate the interactions between the MXene and PVDF-TrFE molecules, molecular dynamics (MD) simulations and density functional theory (DFT) calculations were employed. MXene nanosheets with -OH terminal groups polarize and anchor PVDF-TrFE molecules via hydrogen bonding, resulting in polarization enhancement from 0.56 to 31.41 Debye. By optimizing the MXene concentration, the composite achieved a threefold increase in piezoelectric current output and a nearly eightfold enhancement in low-pressure sensitivity. The unique oriented distribution of MXene and porous structure further enhance the out-of-plane piezoelectric response. For practical application, a wearable sensor was fabricated to detect the pulse waves and finger flexion. With the assistance of deep learning algorithm, bending signals derived from different angles and fingers can be recognized. Additionally, a 3 × 3 sensor array was integrated to monitor the pressure distribution wirelessly on a custom, user-friendly interface. This study provides an efficient, scalable approach to interface piezoelectric composite reinforcement and presents a new perspective on structural design.

## Experimental Section

### Preparation of Ti_3_C_2_T_x_ MXene Nanosheets

The Ti_3_C_2_T_x_ MXene nanosheets was prepared by etching Ti_3_AlC_2_ MAX via LiF/HCl solution as in the previous report, followed by ultrasonic mechanical peeling. In detail, 1.6 g LiF and 40 mL HCl (9 mol L^−1^) were mixed and stirred for 10 min in an ice water bath. Subsequently, 2 g MAX was put into the etching solution and stirred in a 40 °C water bath for 24 h. After that, the suspension was adjusted by deionized water and centrifuged for several times until PH was close to neutral. Ulteriorly, the suspension was treated with an ultrasonication process for 2 h to realize machinal peeling. Finally, the MXene nanosheets water solution was obtained from the middle layer of the solution after centrifugation at 3500 r min^−1^ for 1 h.

### Preparation of MXene/PVDF-TrFE Composite

The MXene nanosheets was collected through filtration and redissolved in DMF/acetone (1 wt%:1 wt%). Subsequently, PVDF-TrFE_80:20_ (Piezotech, Arkema) powder was dissolved in the MXene organic solution with a 10 wt% concentration and stirred for 12 h to obtain a homogeneous state. Prior to blade coating, the MXene/PVDF-TrFE solution was removed bubbles via a negative pressure environment. The MXene/PVDF-TrFE composite film was prepared though blade coating and was dried in a vacuum oven at 40 °C for 24 h. The porous MXene/PVDF-TrFE composite film was prepared though blade coating and was set in air to form shape for 5 min, followed by a water bath treatment in ultrapure water. After the NIPS process, the composite was dried in a vacuum oven at 40 °C for 24 h.

### Fabrication of the Sensor Array

The functional layer consists of nine individual sensors with a sandwich structure and the Ag electrode was made by magnetron sputtering in an argon atmosphere. The PDMS films were prepared by blade coating with a 5:1 weight ratio mixture. Prior to the patterned electrodes were made, the PI marks were needed and fabricated though laser marking with a thickness of 500 μm. The top/bottom Ag electrode and electric lead were fabricated on the PDMS films by magnetron sputtering in an argon atmosphere. The insulation layer was prepared by blade coating and the bond to the top/bottom packing layer was realized by swelling method. Connecting the sensor array to the SCM using a combination of conductive carbon cloth and copper wire. Wireless signal transmission was realized by Bluetooth module.

### Deep Learning for Finger Flexion Recognition

The one-dimensional convolutional neural network (1D-CNN) was constructed in three layers for data feature extraction and input signal recognition. In detail, each type of signal was collected in 10 times, where the length of each record is 1000. The database was randomly divided into three sets at a ratio of 8:1:1, including training set, validation set and testing set. 100 training periods were employed to achieve the signal recognition, and the evolution of the classification accuracy, training accuracy, and training loss were recorded to evaluate the results of deep learning.

### Characterization of Materials

The morphologies and composition of composites were characterized by scanning electron microscopy (JSM-7800F). The porous structure was characterized by an automatic mercury intrusion porosimeter (MIP, MicroActiveAutoPoreV9600). The morphologies and particle size of nanosheets were characterized by transmission electron microscopy (TEM, JEM-2100F), atomic force microscope (AFM, Multimode 8, Bruker), the probe (SCM-PIT-V2, Bruker) was coated with Pt/Ir, and laser particle size analyzer (LPA, Litesizer 500). The crystal structures were characterized by the X-ray diffraction (XRD, Cu as Kα_1_, *λ*≈1.54 Å, PANaltical). The chemical structures of nanosheets were verified by X-ray photoelectron spectroscopy (XPS, Monochromatic X-ray source, Al Kα, 1486.6 eV, Thermo Fisher Scientific K-Alpha). The mechanical performance of composite was measured using a tensile tester machine (LDW-1, Songdu Instrument) at a tensile rate of 10 mm per minute and a puncture rate of 50 mm per minute. The dielectric properties of composite were characterized by a broad dielectric spectrometer (Concept 80, Novocontrol).

## Results and Discussion

### Materials Design

**Fig. 1:**
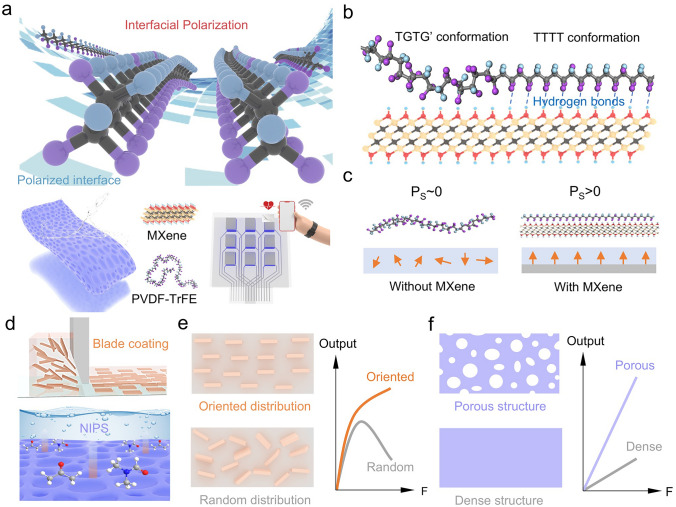
Concept and design of MXene/PVDF-TrFE (MX-P) piezoelectric composite film for pressure sensing. **a** Schematic illustration of the interfacial polarization via the polarized interface and the piezoelectric composite realizing wireless pressure signal detection and recognition. **b** Schematic illustration of the hydrogen bonding between MXene and PVDF-TrFE molecular chain, which benefits the existence of *all-trans* (TTTT) conformation. **c** Schematic illustration of the interfacial polarization via the interaction between MXene and PVDF-TrFE molecular chain. **d** Schematic illustration of oriented distribution and porous structure in composite constructed by NIPS and blade coating. Schematic illustration of **e** oriented strategy and **f** porous structure profiting the enhancement of composite’s electric output

Interfacial polarization is a critical pathway to realize polarization enhancement, which is conducive to enhance piezoelectric response. To construct it, a large scale of polarized interface is indispensable, where MXene is an ideal candidate. Through combining blade coating and NIPS, MXene nanosheets and PVDF-TrFE formed the MXene/PVDF-TrFE piezoelectric composite, which has been employed to realize wireless pressure sensing (Fig. 1a). The MXene nanosheets exhibits the characteristics of two-dimensional materials via LiF/HCl solution etching and have a uniform sheet size (Fig. S1). According to previous studies [36], there is plenty of polar function groups (-OH, -F) on the surface of MXene nanosheets, which provide structural basis to interact with C-F moiety of PVDF-TrFE molecule. The interactions between MXene and molecule, such as hydrogen bonding, promote the conformational transformation of the polymer chains from TGTG’ to TTTT (Fig. 1b), leading to the interfacial polarization and dipole alignment in the out-of-plane direction (Fig. 1c). It is considered to be conducive to introduce structural design for the improvement of piezoelectric response. Thus, blade coating and NIPS were utilized to construct MXene oriented distribution and porous structure (Fig. 1d), respectively. The oriented distribution of MXene prevents the interconnection of MXene nanosheets from forming conductive paths, which is beneficial for exhibiting piezoelectricity under high pressure. Comparing to dense structure, porous structure is benefited from stress concentration to elevate the sensitivity and piezoelectric response of composite.

### MD Simulations of the Interfacial Interactions

**Fig. 2:**
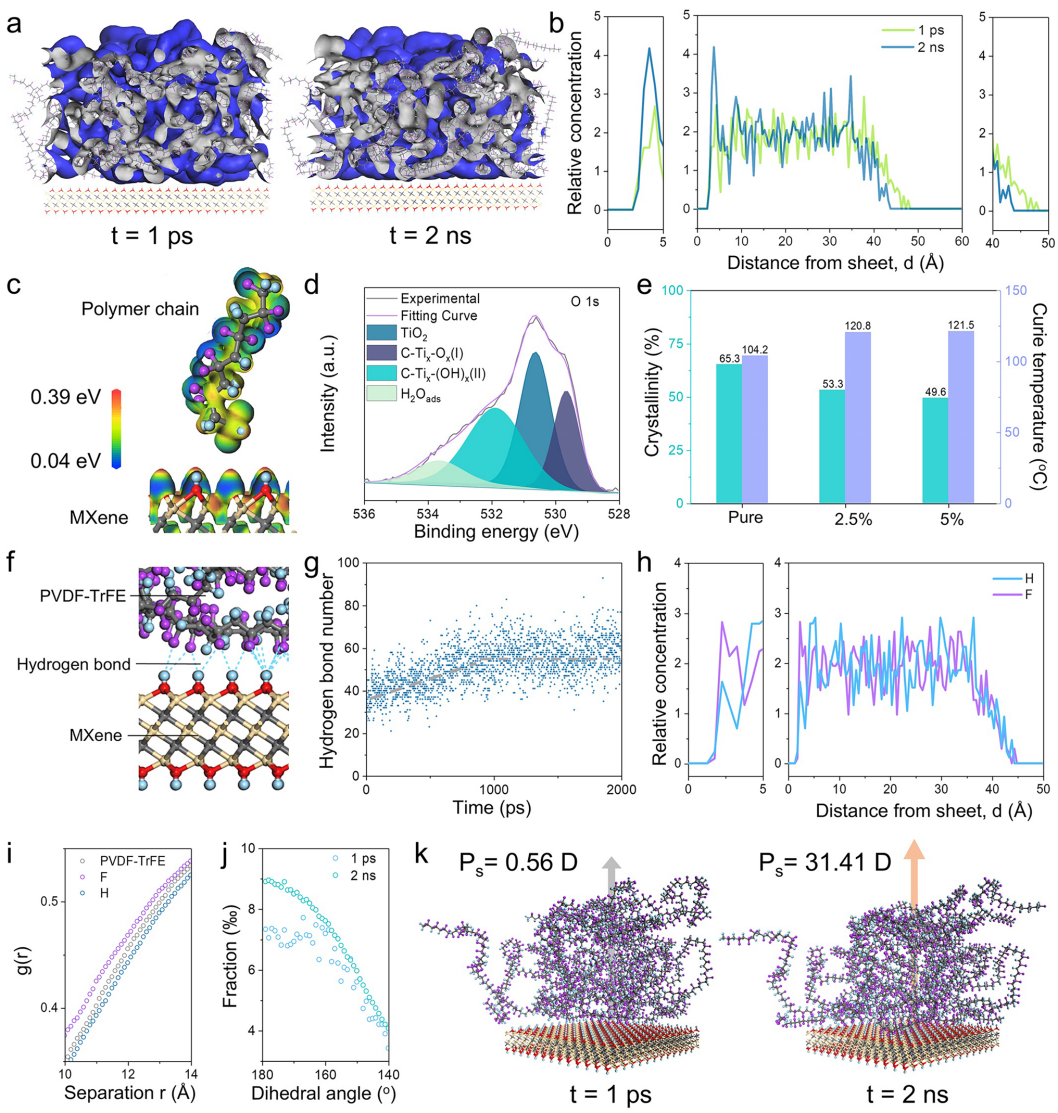
MD simulations of PVDF-TrFE molecular chains polarization on MXene nanosheets. **a** Density distribution snapshots of molecular chains on the surface of MXene nanosheets at 1 ps and 2 ns. **b** Relative concentration distribution function of molecular chains extracted from **a**. **c** DFT calculations of a single PVDF-TrFE chain on the surface of MXene nanosheets with OH termination. **d** O 1* s* XPS spectra of MXene nanosheets. **e** Crystallinity and Curie temperature of composite films with MXene concentrations of 2.5 wt% and 5 wt%. **f** Snapshot for MD simulations of hydrogen bonds at the interface between MXene and PVDF-TrFE. **g** Evolution spectrum of hydrogen bonds number in 2 ns. **h** Relative concentration distribution functions of H and F element on the surface of MXene nanosheets. **i** Localized magnification of the radial distribution function at 10–14 Å for PVDF, H and F elements on the surface of MXene nanosheets. **j** Dihedral angles distribution of PVDF-TrFE chains near the 180° (all-trans) at 1 ps and averaged over 2 ns. **k** Initial (t = 1 ps) and final (t = 2 ns) snapshots for MD simulations of the polarization of PVDF-TrFE at the interface

For having a comprehensive understanding of the interactions between MXene and PVDF-TrFE molecular chains, MD simulations were implemented though a periodic lattice of MXene with -OH surface terminations and 50 “mer” chains of PVDF-TrFE (details information of the MD simulations are discussed in **Note S1**). After 2 ns interaction, the density distribution of polymer has an obvious drift toward the MXene substrate compared to the initial state (Figs. 2a and **S2**). To obtain more details, the relative concentration distributions of the polymer at the 1 ps and 2 ns were analyzed and compared (Fig. 2b). Compared to the initial state, the distribution function at 2 ns has a higher concentration near the substrate and ends closer. Apparently, the interactions draw the polymer chains to assemble on the surface of substrate and leads to a tighter distribution. To confirm this kind of spontaneous aggregation occurs only in the out-of-plane direction, the relative concentration of polymer in other two directions were also analyzed (Fig. S2). During the interaction period, the relative concentration distribution of the polymer shows hardly any variation in the in-plane direction, from which it could be concluded that the interactions at the interface exhibiting a characteristic with a vertical orientation.

The density functional calculations (details information of DFT calculations are discussed in **Note S2**) provide further evidence to confirm the interactions between MXene nanosheets and PVDF-TrFE polymer chains (Figs. 2c and **S3**). Influenced by the polar surface of MXene, the PVDF-TrFE polymer chains undergo partial electron delocalization and transfer. In experiments, it is confirmed that the interfacial interactions have the structural basis based on the surface structure analysis results (Figs. 2d and **S4**) by X-ray photoelectron spectroscopy (XPS). Considering the two-dimensional nature of MXene nanosheets, the interfacial region must have a certain volume inside the composite and it will have an impact on the composite properties. Thus, three composites with different MXene concentrations were prepared and analyzed (Fig. 2e). As presented in Fig. 2e, the Curie temperature increases while the crystallinity decreases with increasing MXene concentration. Apart from interfacial interactions, the nucleation process is also indispensable with the increasing MXene concentration (Fig. S4). However, the nucleation did not appear an evident impact. It is inferred that the substantial interfacial region and the interfacial interactions jointly anchor the polymer chains and restrict the movement of crystallization [37], confirming the success of the interfacial design.

On the basis of above analysis and results, it is reasonable to extrapolate that the hydrogen bonding plays a decisive role in the composition of the interactions. Since that, simulations and analyses were conducted on the evolution of hydrogen bonds during the interaction period (Figs. 2f** and S5, **Movie S1). The hydrogen bonds exist from the initial state and it draws the polymer chains drifting toward the MXene substrate, leading to a further increase in hydrogen bonding until the dynamic equilibrium is met (Fig. 2g). To acquire an exhausting understanding of the interfacial hydrogen bonds, the distribution of the H, F atoms in the polymer chains and the PVDF-TrFE molecule were further investigated as a function of the separation from the MXene substrate (Fig. 2h). It is obvious that the F atoms have a higher concentration on the surface of the MXene substrate relative to the H atoms, from which it can be inferred that the F atoms on the C-F moieties is more likely to form the hydrogen bond with the H atoms on the terminations of the MXene substrate. The radial distribution function (RDF) of the H, F atoms in the polymer chains and the PVDF-TrFE molecule (Figs. 2i and **S5**) also confirm that the F atoms are closer to the MXene substrate compared to the H atoms.

It is believed that some changes in the properties of polymers occur as a result of interfacial interactions. There are four main dihedral angles exhibited in the PVDF-TrFE polymer chains, ± 180° (*trans,* T) and ± 60° (*gauche,* G), respectively. The *trans*-*gauche* (TGTG’) conformation is thermodynamically preferred, while the all-*trans* (TTTT) conformation has a stronger dipole moment. Therefore, the fraction of dihedral angles can reflect the local electroactivity change of the PVDF-TrFE molecule adjacent to the MXene substrate. After the 2 ns equilibrium process, the dihedral angles possess a higher proportion near the 180°, indicating the conformation transition from *gauche* to *trans* (Fig. 2j) as a consequence of interfacial interactions. In addition, the interfacial interactions promote the out-of-plane net spontaneous polarization from 0.56 to 31.41 Debye (Fig. 2k) through the equilibrium process, while the in-plane net spontaneous polarization process changes weakly (**Fig. S6**). Ulteriorly, after 2 ns interaction, an extreme electric field 1 V nm^−1^ was applied to the system for another 2 ns. The atomic distributions and dihedral angles of the polymer chains show slight changes (**Fig. S7**), validating the critical role of interfacial molecular polarization via the interfacial interactions, despite the enhancement of the net spontaneous polarization that can be attributed to the atomic or electron polarization.

### Structural Characterization and Mechanism Analysis of Piezoelectric Composites

**Fig. 3:**
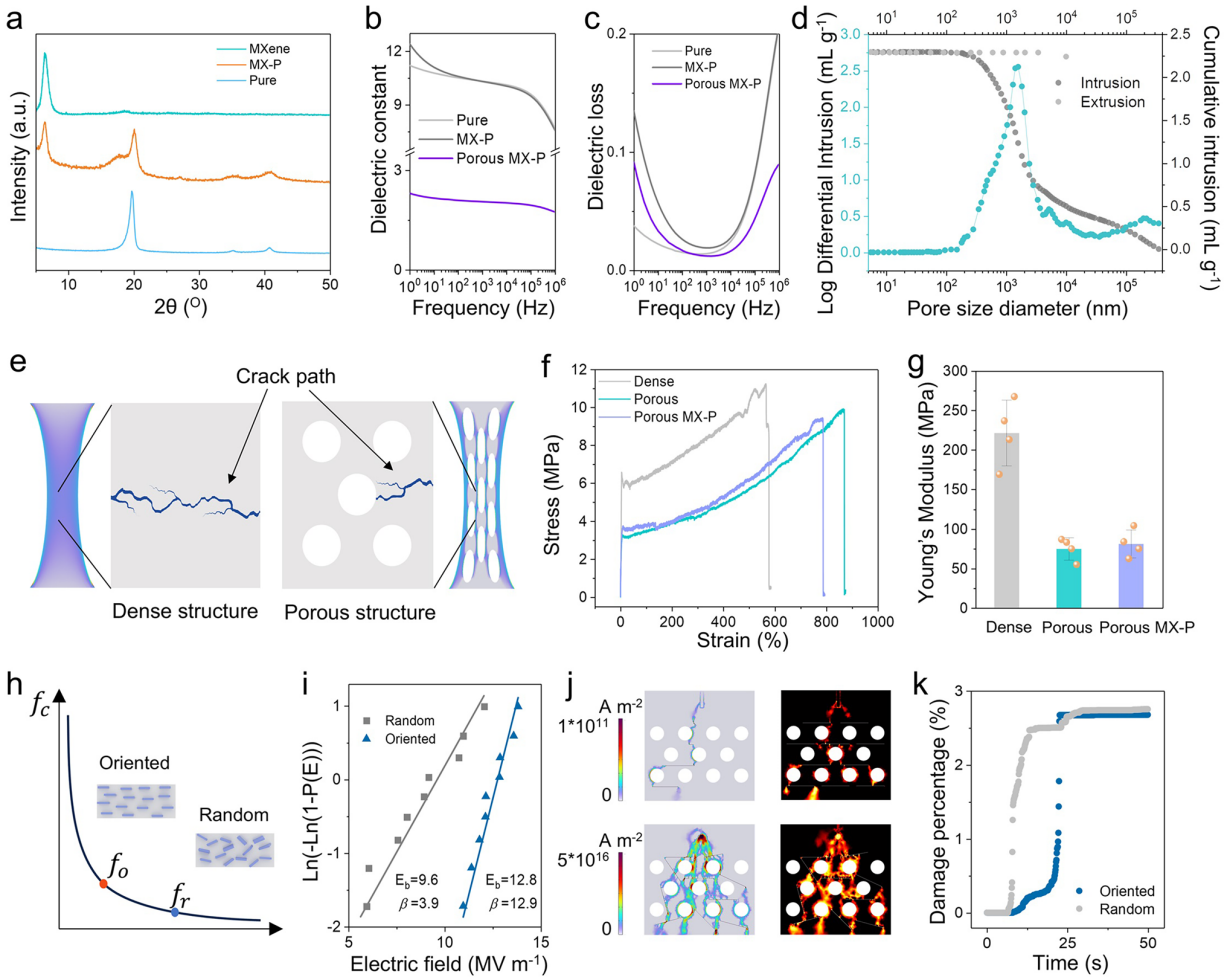
Characterization of materials and mechanisms analysis. **a** XRD spectra of MXene, pure PVDF-TrFE and MX-P. **b** Frequency-dependent dielectric permittivity and **c** dielectric loss of pure PVDF-TrFE, MX-P and porous MX-P. **d** Intrusion and extrusion mercury curve and pore size distribution curve of porous MX-P films. **e** Schematic illustration of porous structure preventing further crack growth. **f** Stress–strain curves of dense, porous PVDF-TrFE and porous MX-P. **g** Young’s modulus of dense, porous PVDF-TrFE and porous MX-P. Error bars are standard deviations derived from four parallel tests. **h** Schematic illustration of nanofiller distribution’s impact on the percolation threshold. **i** Weibull distribution analysis of the breakdown strength of MX-P composites with different MXene distribution. **j** Simulative current paths of dielectric breakdown (left) and failure phase distribution (right) for MX-P composites with oriented (top) and random (bottom) MXene distribution. **k** Percentage of tissue damaged by dielectric breakdown as a function of time for MX-P composites with different MXene distribution

Based on the interfacial polarization strategy, the MXene/PVDF-TrFE (MX-P) piezoelectric composites were well-prepared via blade coating and NIPS. The as-prepared composite films have the structural and chemical characteristics of MXene and PVDF-TrFE, as evidenced by the results of X-rays diffraction (XRD) (Fig. 3a) and energy dispersive X-ray spectroscopy (EDS) (**Fig. S8**). During the NIPS, the solvent exchange process is very intense, leaving lots of exchange channels that form the porous structure. According to the following Maxwell–Garnett (M-G) formula, the dielectric constant of the composite will decrease significantly as the porous structure is being implemented, which has been validated by the results of dielectric measurements (Fig. 3b).

1 $${\varepsilon =\varepsilon }_{m}\left(\frac{{\varepsilon }_{i}+2{\varepsilon }_{m}+2f\left({\varepsilon }_{i}-{\varepsilon }_{m}\right)}{{\varepsilon }_{i}+2{\varepsilon }_{m}-f\left({\varepsilon }_{i}-{\varepsilon }_{m}\right)}\right)$$

In the M-G formula, the *ε*, *ε*_*m*_, *ε*_*i*_ and *f* represent the dielectric constant of composite, the dielectric constant of the matrix, the dielectric constant of the additive and the volume of the additive, respectively. It is noteworthy that both the dielectric constant and the dielectric loss of MX-P as a function of frequency occur a lift below 10^3^ Hz (Fig. 3b, c), which can be attributed to the interfacial polarization. To further investigate the porous structure, the scanning electron microscope (SEM) and the automatic mercury intrusion porosimeter (MIP) were applied to obtain more detailed structural information (Figs. 3d and **S9**). The composite possesses relatively uniform pore size and distribution, and average pore diameter is 1186.76 nm (detailed information on the MIP measurement results are presented in Table S1). It is believed that the porous structure can disperse stress concentration of crack tip and impede the growth of cracks (Fig. 3e). Thus, the stress–strain curve of porous MX-P was measured (**Fig. S10**) and compared to the porous PVDF-TrFE and the dense PVDF-TrFE (Fig. 3f). It is found that the porous structure evidently improves the elongation at break of the materials, which is consistent with the previous conjecture. In addition, the introduction of the porous structure visibly reduces the Young’s modulus of the composite that benefits the composite in terms of improved flexibility.

Percolation is widely present in composite materials, where the properties of materials occur dramatic changes. Instead of a linear rule, the properties of composite can be described by the following formula:

2 $$Properties\propto {\left|f-{f}_{c}\right|}^{\pm e}$$

where *f*, *f*_*c*_ and *e* represent the volume fraction, percolation threshold and critical exponent [38]. Considering the conductivity of MXene nanosheets, it is necessary for the composite to improve the percolation threshold to reduce the possibility of nanosheets bridging. Therefore, the oriented distribution of MXene nanosheets was implemented via blade coating [39], which can improve the maximum MXene addition concentration and further enlarge the interfacial region (Fig. 3h). Compared to the random distribution, the oriented distribution enhances the dielectric breakdown strength (Fig. 3i and details of dielectric breakdown measurement are presented in **Note S3**), i.e., diminishes the possibility of bridging. The effect of oriented distribution on dielectric breakdown strength has also been validated by simulation results (Fig. 3j, Movie S2 and details of dielectric breakdown simulations are presented in **Note S4**). At the same electric field strength, there are more breakdown pathways in the composite with a random nanosheet distribution than in that with an oriented distribution, leading to more irreversible transition region (orange region in Fig. 3j right channel) caused by accumulated heat (**Fig. S11**). In addition, oriented nanosheet composites exhibit delayed progression to equivalent damage levels under electric stress, indicating improved resistance to partial discharge propagation.

### Sensing Performance of Piezoelectric Composite

**Fig. 4:**
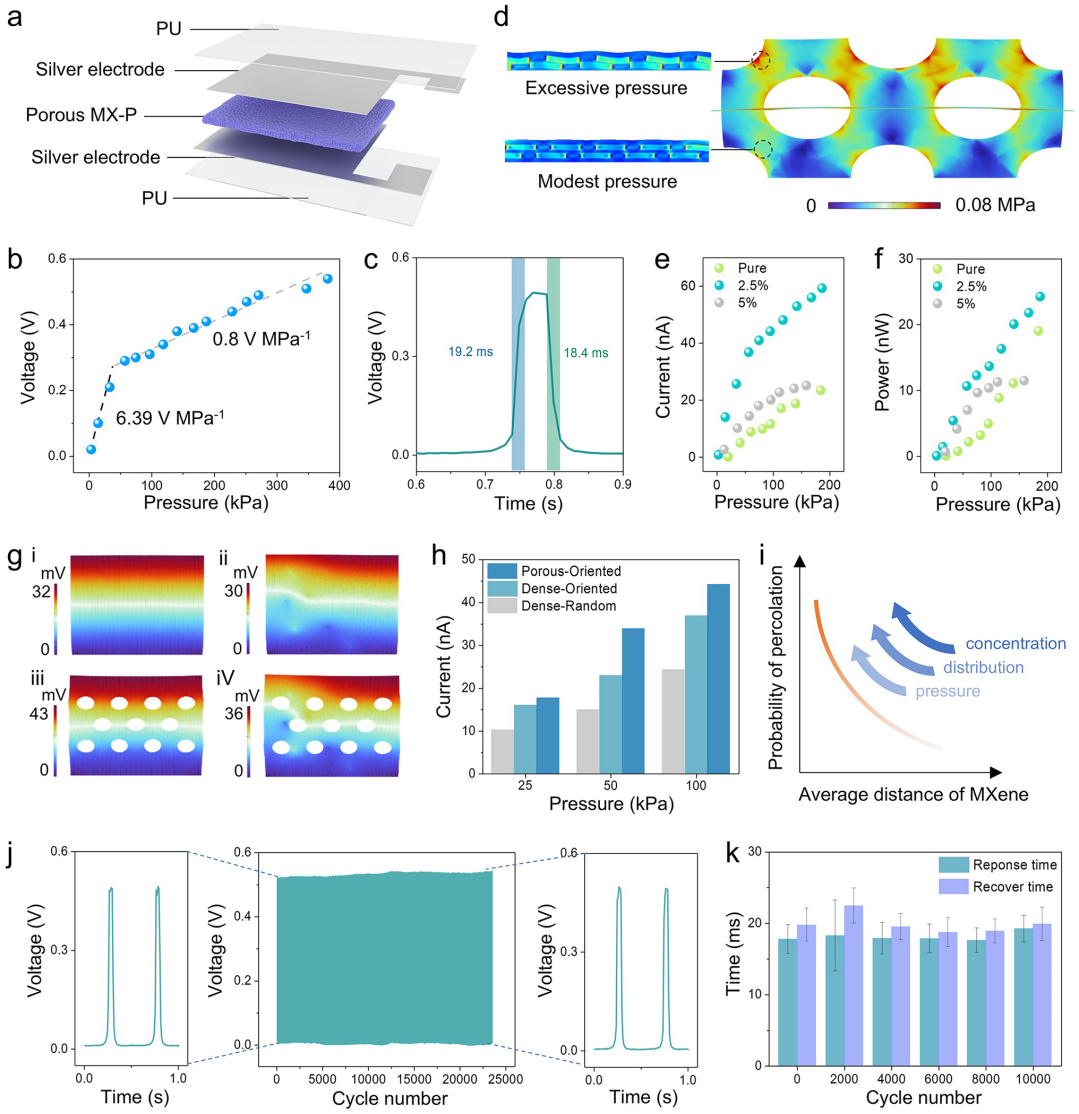
Electrical properties and mechanism of porous MX-P-based piezoelectric sensor. **a** Schematic illustration of piezoelectric sensor in explosive view. **b** Voltage output of piezoelectric sensor under different pressure. **c** Response and recover time of piezoelectric sensor. **d** Vertical sections for simulating pressure distribution in porous films under 50 kPa pressure and diagram illustration of MXene nanosheets at different pressure zones. **e**, **f** Electric output of piezoelectric sensors with different MXene contents. **g** Simulations of electric potential distribution under pressure in dense MX-P for (**i**) MXene oriented distribution, (**ii**) MXene random distribution and in porous MX-P for (**iii**) MXene oriented distribution, (**iv**) MXene random distribution. **h** Comparative electric output of composites with different structures. Dense-random, dense-oriented and porous-oriented represent dense MX-P with MXene random distribution, dense MX-P with MXene oriented distribution and porous MX-P with MXene oriented distribution, respectively. **i** Schematic illustration of pressure, MXene concentration and distribution’s impacts on electric output. **j** Durability test of piezoelectric sensor under a 5 N force. **k** Evolution of sensor’s response capability during the durability test

To investigate the performance of the porous MX-P composite under external stimulation, the composite was fabricated into a sandwich-structured device (Fig. 4a) and the measurements were conducted on a home-made platform (**Fig. S12**). The porous MX-P composite possesses typical piezoelectric outputs, but is 8 times more sensitive in low-pressure region than in other regions (Fig. 4b). Besides, the device has a favorable response and recovery performance under the external stimulation (Fig. 4c). The piezoelectric output of the composite in this work has an obvious enhancement as compared to the composites in the previous work (Table S2). To investigate the high sensitivity of the low-pressure region the porous structure was implemented in simulations for its behavior under external pressure (Figs. 4d and **S13**). Under the external pressure, local stress concentration occurs in the material where the material withstands higher pressure and saturate earlier. However, the average spacing of MXene nanosheets would decrease rapidly in the stress concentration region, leading to the local concentration of MXene exceeding the percolation threshold. In order to explore the effect of the MXene concentration on performance, the outputs of composites with different MXene concentrations were measured and compared (Fig. 4e, f). It is clear that the addition of MXene improves the electric outputs (the output current of 2.5 wt% is almost 3 times that of the pure one at 200 kPa), while the effect was weakened once the content over the percolation threshold. The above results validate the effect of interfacial polarization and benefits of oriented distribution and importance of proper addition concentration.

To further investigate the impacts of structural design on performance, both simulations and experiments were utilized to compare the electric outputs of composites with/without structural design. From the simulation results, it can be found that the porous structure effectively improves the electrical performance, while the MXene random distribution can play a negative role (Figs. 4g and **S14**). The same conclusion can be acquired in the experiment results by comparing the electric outputs of composites (Figs. 4h and **S15**). In fact, the abundant polar functional groups give MXene a polar surface but also a conductive property that should be considered in enhancing piezoelectricity of the composite. The bridging of MXene nanosheets is detrimental to the piezoelectric outputs of composites (**Fig. S15**). The design of MXene orientation can effectively inhibit the occurrence of bridging phenomenon, which enables the composite films to have higher MXene additions and more significant interfacial polarization effects, and ultimately enhances the electrical output of the materials. Based on the above results, it can be concluded that the controlling average distance of MXene nanosheets plays a critical role in preventing percolation, which is mainly affected by MXene concentration, MXene distribution and external pressure (Fig. 4i). Through controlling the addition concentration and introducing oriented distribution structure, the piezoelectric output of porous MX-P composite possesses long-term use stability (Fig. 4j, k).

### Pressure Sensing for Exploring Potential Applications

Attributed to the inherent characteristics of piezoelectricity, the piezoelectric sensor has excellent response to dynamic stress, which occurs very frequently in the human body [40]. Despite MXene and PVDF-TrFE are both biocompatible materials, it is still necessary to validate the biological harmlessness of composite. Fortunately, the porous MX-P composite did not present evidently negative effect on the cell growth and survival. In addition, the high porosity of the porous MX-P composite imparts excellent moisture permeability, which is critical for wearing comfort (**Figs. S16** and **S17**).

**Fig. 5:**
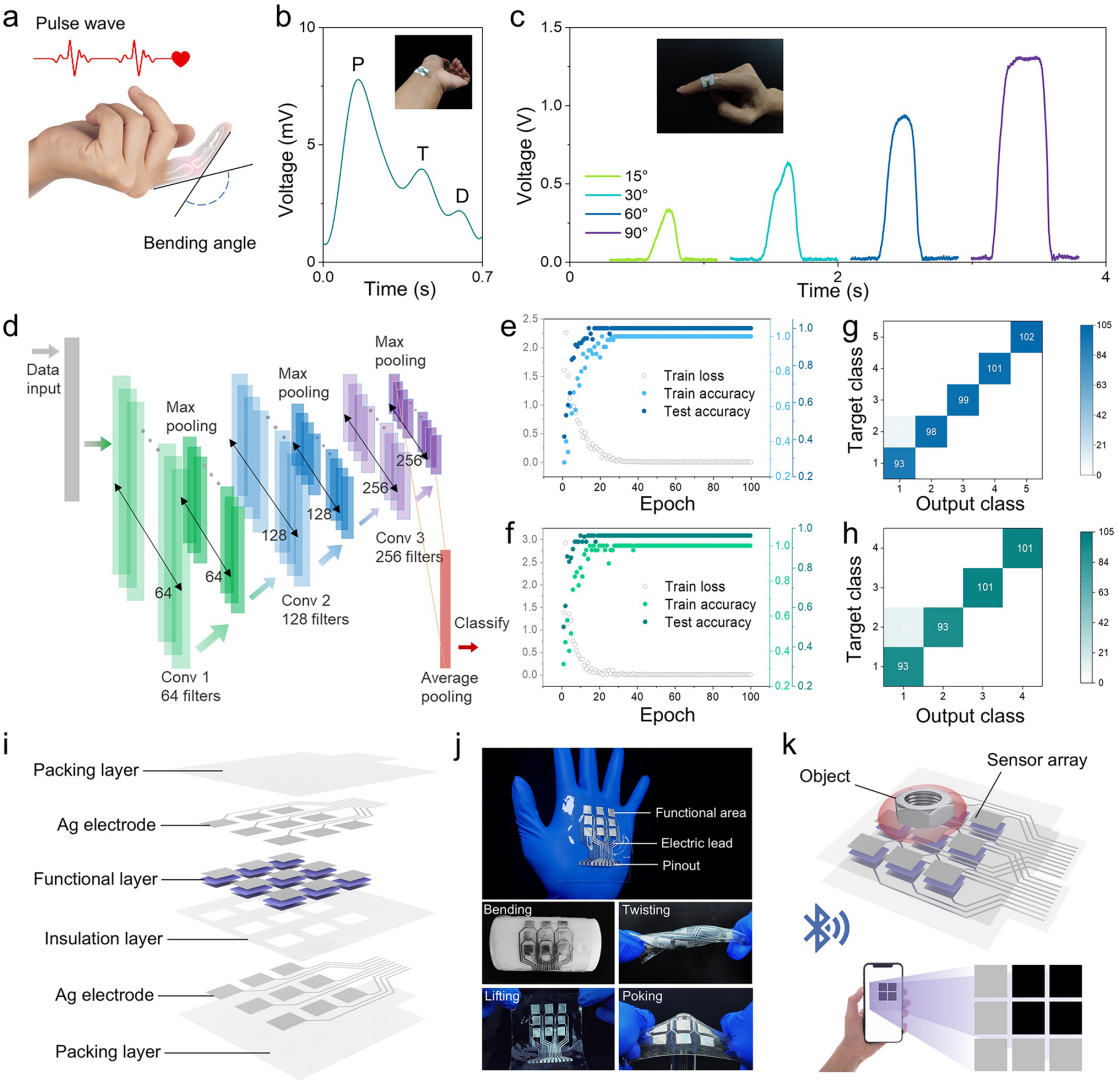
Physiological signal monitoring and application of sensor array. **a** Schematic diagram of detecting artery pulse waves and finger flexion with sensors. **b** Radial artery pulse signal detected in one cardiac cycle. Inset digital image presents the scene of artery pulse detection. **c** Comparative pulse signals generated by different degrees of finger flexion. Inset digital image presents the scene of finger flexion detection. **d** Detailed architecture diagram of constructed 1D-CNN model. **e** Evaluation of classification, train accuracy and loss function in 100 epochs for five finger flexion. **f** Evaluation of classification, train accuracy and loss function in 100 epochs for four kinds of bending degrees. **g** Confusion matrix for five finger flexion. **h** Confusion matrix for four kinds of bending degrees. **i** Schematic diagram of the sensor array in explosive view. **j** Photographic images showing detailed structure and mechanical compliance of sensor array. **k** Schematic diagram of wireless pressure detection by sensor array

Artery pulse and finger flexion (Fig. 5a) are two of the most common dynamic stress sources that can deliver information about health [41]. Owing to the high sensitivity of porous MX-P in the low-pressure region, the sensor can successfully capture the signal of pulse wave and distinguish the physiological characteristics, including percussion wave (P), tidal wave (T), dicrotic wave (D) (Figs. 5b and **S17**). Besides, the signals from finger flexion can also be detected by the senor, and the signals derived from different bending angles are different (Fig. 5c). Attributed to the deep learning algorithm (Fig. 5d), the signals of five finger flexion at different angles can be distinguished. The proposed models present excellent classification accuracy and robustness through training process (Fig. 5e, f). Furthermore, the system can distinguish the signals from different fingers with an accuracy of 99% and different bending angles with an accuracy of 96.25% (Fig. 5g, h). Based on the excellent performance in pressure sensing, nine sensors were integrated into a 3 × 3 sensor array, exploring the potential applications in sensing pressure distribution. In detail, the sensor arrays are fabricated based on a sandwich construction of individual sensors and are combined with polydimethylsiloxane (PDMS) films that have a circuit design, as well as an insulation layer that prevents contact between the top and bottom electrical conductors (Figs. 5i and **S18**). Benefiting from the flexible substrate, excellent flexibility and tolerance to various deformations including bending, twisting, lifting and poking, were present by the sensor array (Fig. 5j). Assisted by the single chip microcomputer (SCM) and customized display terminal (Figs. 5k and **S18**), the system possesses the ability to monitor the pressure in real-time with well resolution (**Fig. S19** and Movie S3).

## Conclusions

In conclusion, we designed a dual structural reinforced MXene/PVDF-TrFE piezoelectric composite with MXene oriented distribution and porous structure to realize the enhancement of piezoelectricity. Based on the MD simulations, DFT calculations, and experiments, the detailed information of the polarization improvement arising from the interfacial interactions and the enhancement on the piezoelectricity of the composite was clearly revealed. The MXene oriented distribution realized by blade coating decreases the possibility of forming conductive path and increases the fraction of interface. Furthermore, the porous structure endows the composite with excellent flexibility, high sensitivity in low-pressure region and high piezoelectric response. On the basis of above properties, the piezoelectric sensor was fabricated to detect the artery pulse and finger flexion, of which the signals can be recognized with the assistance of deep learning algorithm. Finally, a 3 × 3 sensor array was integrated to explore the potential application in pressure distribution. It is believed that this work provides an efficient, scalable approach to interface piezoelectric material reinforcement and presents a new perspective on structural design.

## Supplementary Information

Below is the link to the electronic supplementary material.

Supplementary file1 (MP4 19626 KB)

Supplementary file2 (MP4 1995 KB)

Supplementary file3 (MP4 17092 KB)

Supplementary file4 (DOCX 6441 KB)
