# Supplementary material for: Dual Structure Reinforces Interfacial Polarized MXene/PVDF-TrFE Piezoelectric Nanocomposite for Pressure Monitoring
Source: Nanomicro Lett. 2025 Jul 4;17:320. doi: 10.1007/s40820-025-01839-5 (PMC12227402; doi:10.1007/s40820-025-01839-5)
Supplement: Supplementary file 4 — Supplementary file4 (DOCX 6441 KB) [file 40820_2025_1839_MOESM4_ESM.docx]

Supporting Information for

**Dual-Structure Reinforces Interfacial Polarized MXene/PVDF-TrFE Piezoelectric Nanocomposite for Pressure Monitoring**

Yong Ao^1^, Long Jin^1,^*, Shenglong Wang^1^, Bolin Lan^1^, Guo Tian^1^, Tianpei Xu^1^, Longchao Huang^1^, Zihan Wang^1^, Yue Sun^1^, Tao Yang^1^, Weili Deng^1^, Fan Yang^2,3,^*, Weiqing Yang^1,2,^*

^1^ Key Laboratory of Advanced Technologies of Materials, Ministry of Education, School of Materials Science and Engineering, Southwest Jiaotong University, Chengdu 610031, P. R. China

^2^ Research Institute of Frontier Science, Southwest Jiaotong University, Chengdu 610031, P. R. China

^3^ Shanghai Institute of Traumatology and Orthopaedics, Ruijin Hospital, Shanghai Jiao Tong University School of Medicine, Shanghai 200025, P. R. China

*Corresponding authors. E-mail: [longjin@swjtu.edu.cn](mailto:longjin@swjtu.edu.cn) (Long Jin); [yf12498@sjtu.edu.cn](mailto:yf12498@sjtu.edu.cn) (Fan Yang); [wqyang@swjtu.edu.cn](mailto:wqyang@swjtu.edu.cn) (Weiqing Yang)

**Note S1** **Molecular dynamics (MD) simulations**

In the MD simulations, 14 PVDF-TrFE molecular chains and one MXene nanosheet with -OH surface terminations form the model, having an initial polymer density of 1.3 g cm^-3^. Each PVDF-TrFE molecular chain contains 10 TrFE and 40 VDF monomers, corresponding to molar concentrations of 20 mol% TrFE and 80 mol% VDF. The atomic partial charges of PVDF-TrFE molecular chains and MXene are adopted from the first-principal calculations. For equilibration, the polymer chains and MXene nanosheet are initially geometry optimized and energy minimized. The Universal Force Field is utilized to describe the interactions of the PVDF-TrFE chains and MXene nanosheet. For equilibration, the model is simulated in the NVT (constant number of atoms, volume, and temperature) ensemble at 298 K for first 2 ns and under an electrical field of 1 V nm^-1^ for another 2 ns. The Newton’s equations of motion were integrated using the velocity-Verlet algorithm with a time step of 1 fs. Temperature and distribution of atomic velocities in a system are related through the Maxwell-Boltzmann equation. Van der Waals and electrostatic interactions were considered and the cut-off distance of non-bonded van der Waals interactions was set as 15.5 Å. All the MD simulations were carried out using Materials Studio software.

**Note S2 Density functional theory (DFT) calculations**

In the density functional theory calculations, the polymer chains contain 1 TrFE and 4 VDF monomers, corresponding to molar concentrations of 20 mol% TrFE and 80 mol% VDF. The MXene nanosheet supercell with -OH termination of 2×2 lateral size is used. The density functional theory calculations of electrostatic potential are performed using Dmol3 module. The generalized gradient approximation (GGA) and the Becke exchange functional and the Lee-Yang-Parr correlation (BLYP) are used for the exchange-correlation energy. The convergence tolerance of electronic self-consistent energy and force is 1.0×10^-6^ eV∙atom^-1^ and 0.05 eV∙Å^-1^. The adsorption energy (*ΔE*) in H_2_O/PVDF-TrFE model is determined as:

$$\Delta E=E_{H_{2}O/PVDF-TrFE}-E_{H_{2}O}-E_{PVDF-TrFE}$$

where *E_H2O/PVDF-TrFE_*, *E_H2O_* and *E_H2O/PVDF-TrFE_* refer to the total energy of the H_2_O/PVDF-TrFE model, the energy of the H_2_O and the energy of the PVDF-TrFE molecule, respectively. All the calculations are carried out by the Materials Studio software.

**Note S3 Dielectric breakdown measurement**

The dielectric breakdown of MX-P composites is performed via the Dielectric Withstand Voltage Test System at a ramping rate of 500V s^-1^ and a limit current of 5 mA.

The dielectric breakdown behavior of the MX-P composites is analyzed by a two-parameter Weibull distribution function as follows:

$$P\left( E \right)=1-exp(-(E/E_{p})^\beta)$$

Where *E* is the measured breakdown field, *P(E)* is the cumulative probability of electric breakdown at *E*, *E_b_* is the statistical breakdown strength, corresponding to the *P(E)* equals 63.2%. The Weibull parameter *β* is an evaluator of the experimental breakdown field distribution.

**Note S4 Simulations of dielectric breakdown**

The dielectric breakdown behavior is sensitive to temperature according to the breakdown theory proposed by Wagner et al. The breakdown function as follows:

$$E_{b}=\sqrt{\frac{\beta\rho}{0.24Sae}}e^{\frac{-aT_{0}}{2}}$$

Where *β* is the coefficient of heat release, *T_0_* is the initial temperature of system, *S* and *ρ* are the area and resistivity of conductive channel in dielectric, *α* is the coupling coefficient between conductivity and temperature. In this work, the current function in the dielectric is expressed as follows:

$$\nabla J=Q_{j}$$

$$J=\sigma E+\frac{\partial D}{\partial t}+J_{e}$$

Where *J* and *Q_j_* are the flux and electric charge, *σ* and *D* are the conductivity and electric displacement polarization, *J_e_* is the externally generated current density. Furthermore, the thermal function in the dielectric is descripted as follows:

$$\boldsymbol{q}=-d_{z}k\nabla T$$

$$d_{z}\rho C_{p}\frac{\partial T}{\partial t}+d_{z}\rho C_{p}\boldsymbol{\mu}\cdot\nabla T+\nabla\cdot\boldsymbol{q=}d_{z}Q+q_{0}+d_{z}Q_{ted}$$

Where ***q*** is the conductive heat flux, *d_z_* is the thickness of domain in the *z* direction, *k* is the thermal conductivity. *ρ* is density, *C_p_* is the specific heat capacity at constant pressure, ***μ*** is fluid velocity vector, *q_0_* is the inward heat flux and *Q_ted_* is the thermoelastic damping. Moreover, the dielectric will undergo an irreversible phase transition when the temperature exceeds the critical value, causing the conductance of the model to increase sharply. Thus, the association function between conductivity and heat has been considered in the simulation.

**Supplementary Figures**

**
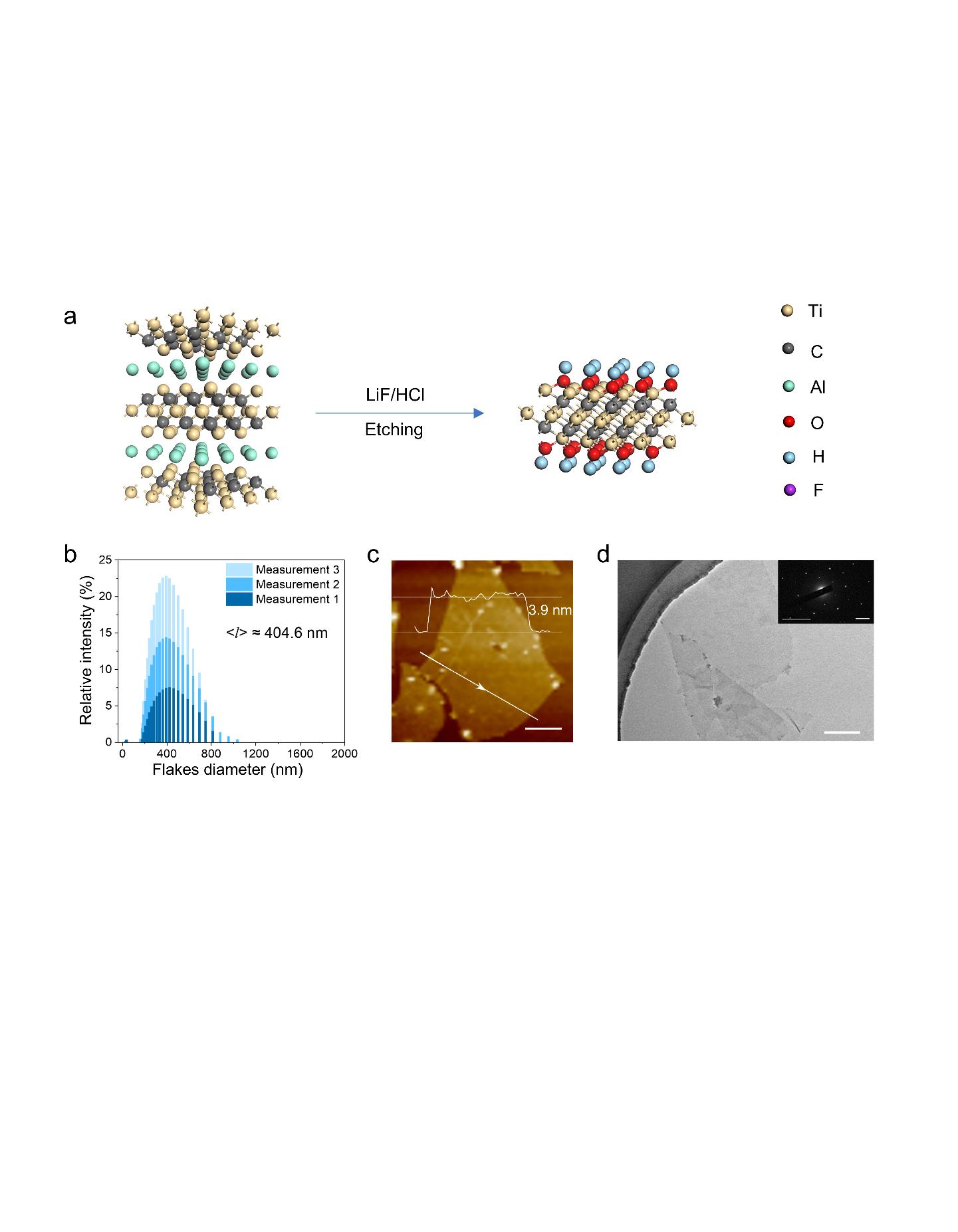
**

**Fig. S1** Preparation and characterization of MXene nanosheets. **a** Schematic illustration of proceeding MXene by LiF/HCl etching method. **b** Particle size distribution of MXene measured by laser particle size analyzer (LPA). **c** AFM image and corresponding height profile along the line of MXene nanosheets. Scale bar is 200 nm. **d** TEM image of MXene nanosheets. Inset shows the selected area electron diffraction (SAED) pattern. Scale bar is 200 nm and 50 nm in the inset

**
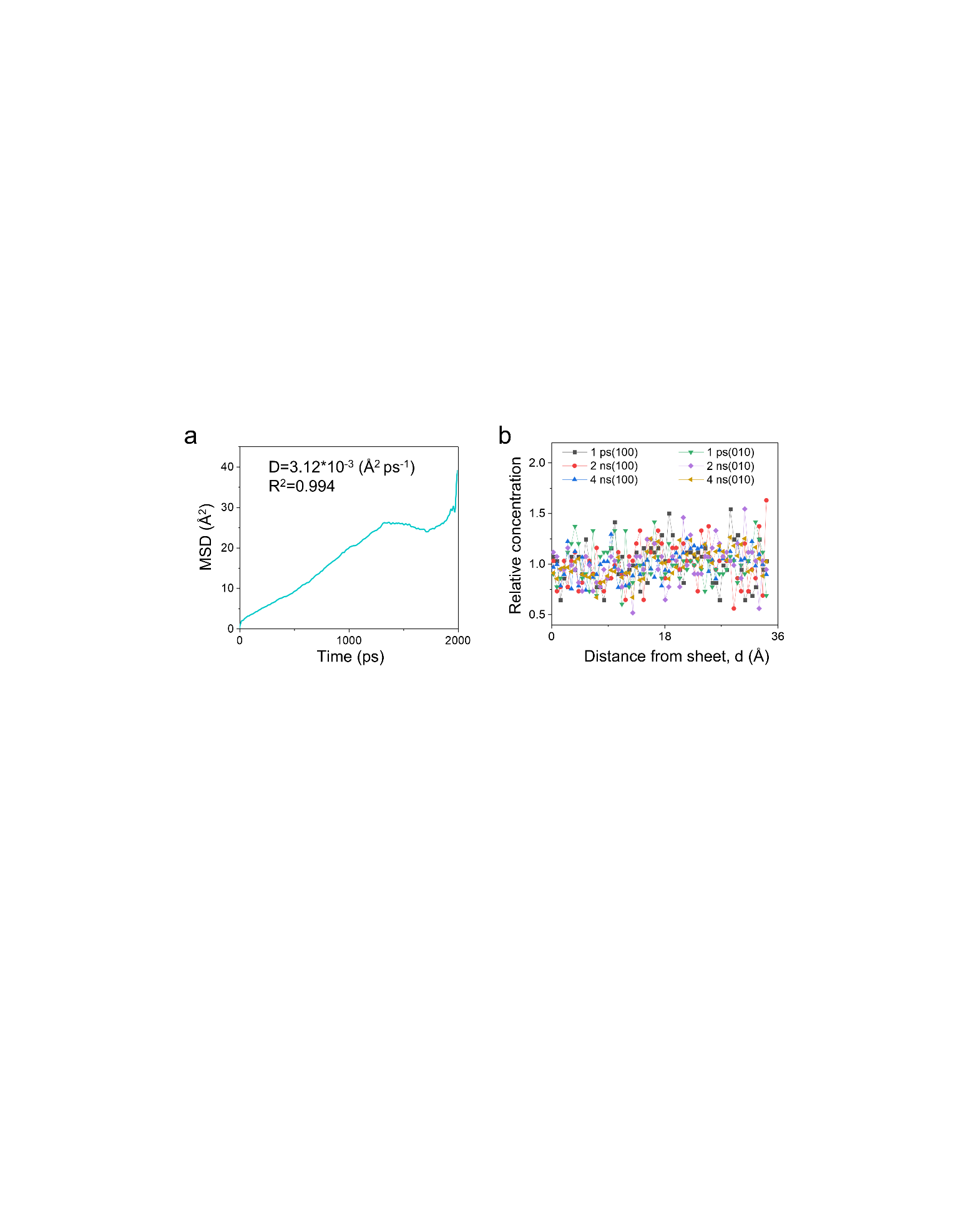
**

**Fig. S2** MD simulations of the overall PVDF-TrFE molecular chains distribution on the surface of MXene flat as a function of time. **a** Mean squared displacement of overall molecular chains in 2 ns. **b** Comparative concentration evolution of overall molecular chains along the [010] and [100] directions

**
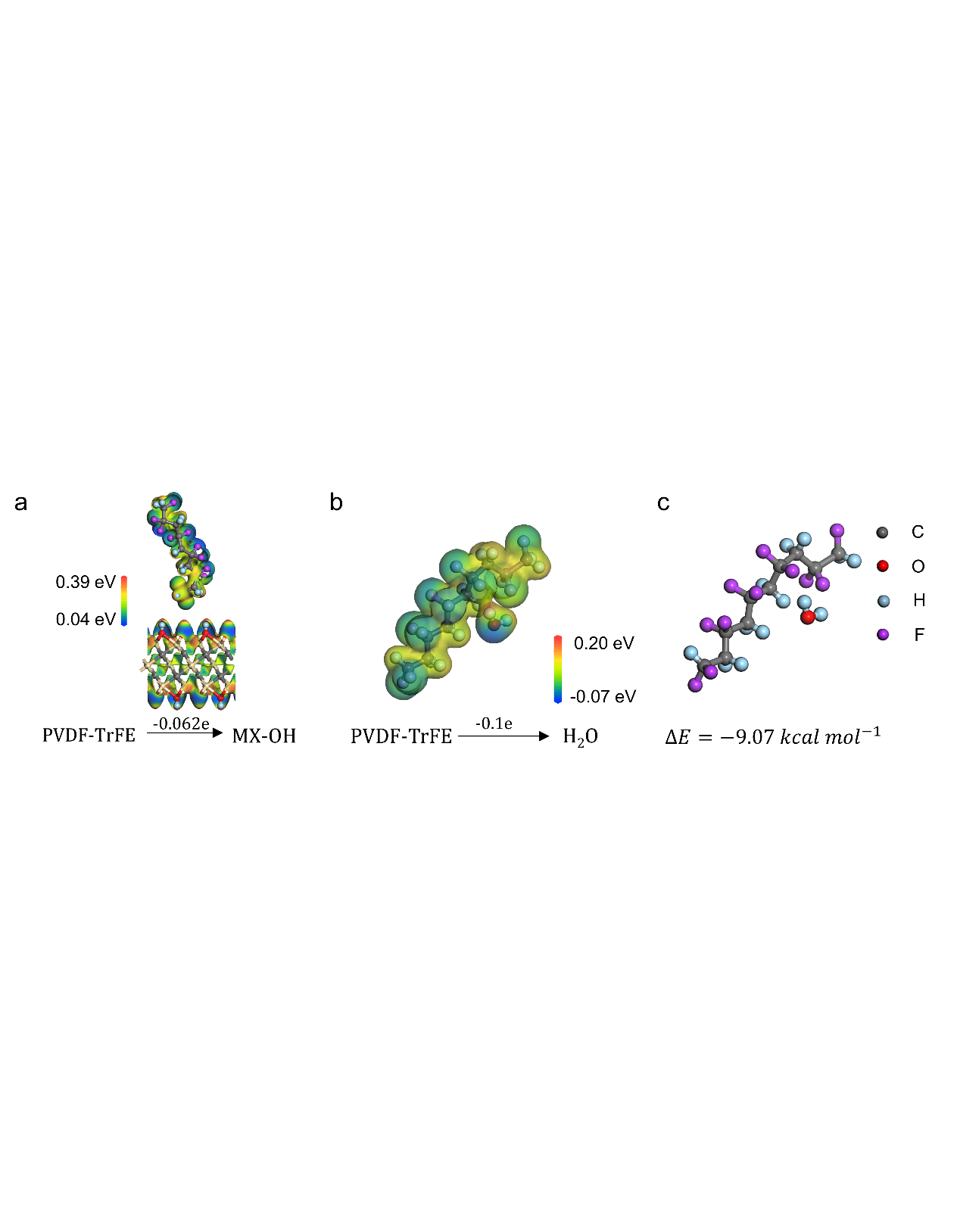
**

**Fig. S3** DFT calculations for the interaction of PVDF-TrFE with MXene and PVDF-TrFE with H_2_O. **a** DFT calculations of potential distribution and electron transfer for MX-P. **b** DFT calculations of potential distribution and electron transfer for H_2_O/PVDF-TrFE. **c** The interaction energy between PVDF-TrFE with H_2_O

**
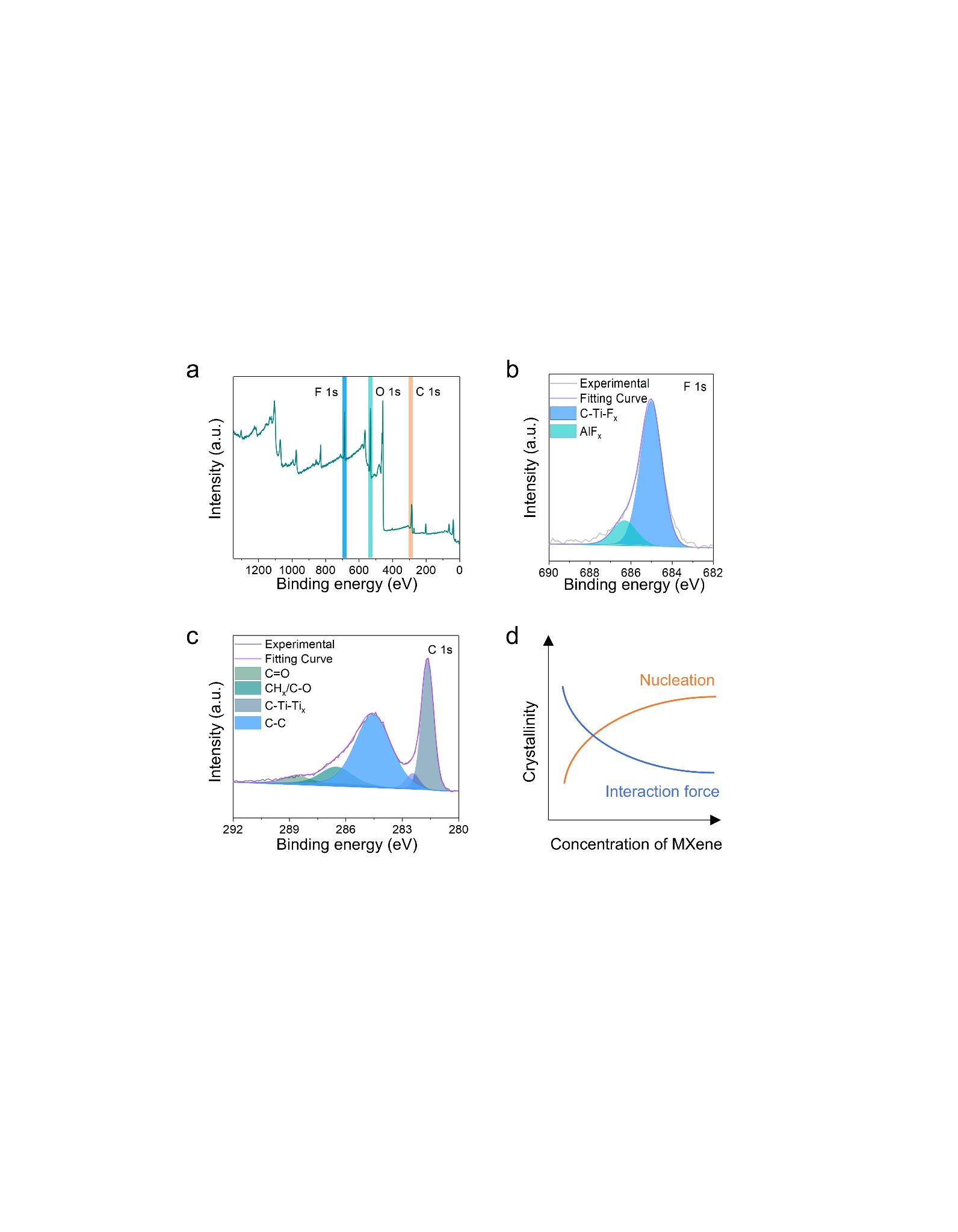
**

**Fig. S4** XPS analysis of MXene and the corresponding binding energy spectra of F and C elements. **a** XPS survey spectra of MXene on a silicon substrate. **b** The deconvoluted F 1s core-level spectrum. **c** The deconvoluted C 1s core-level spectrum

**
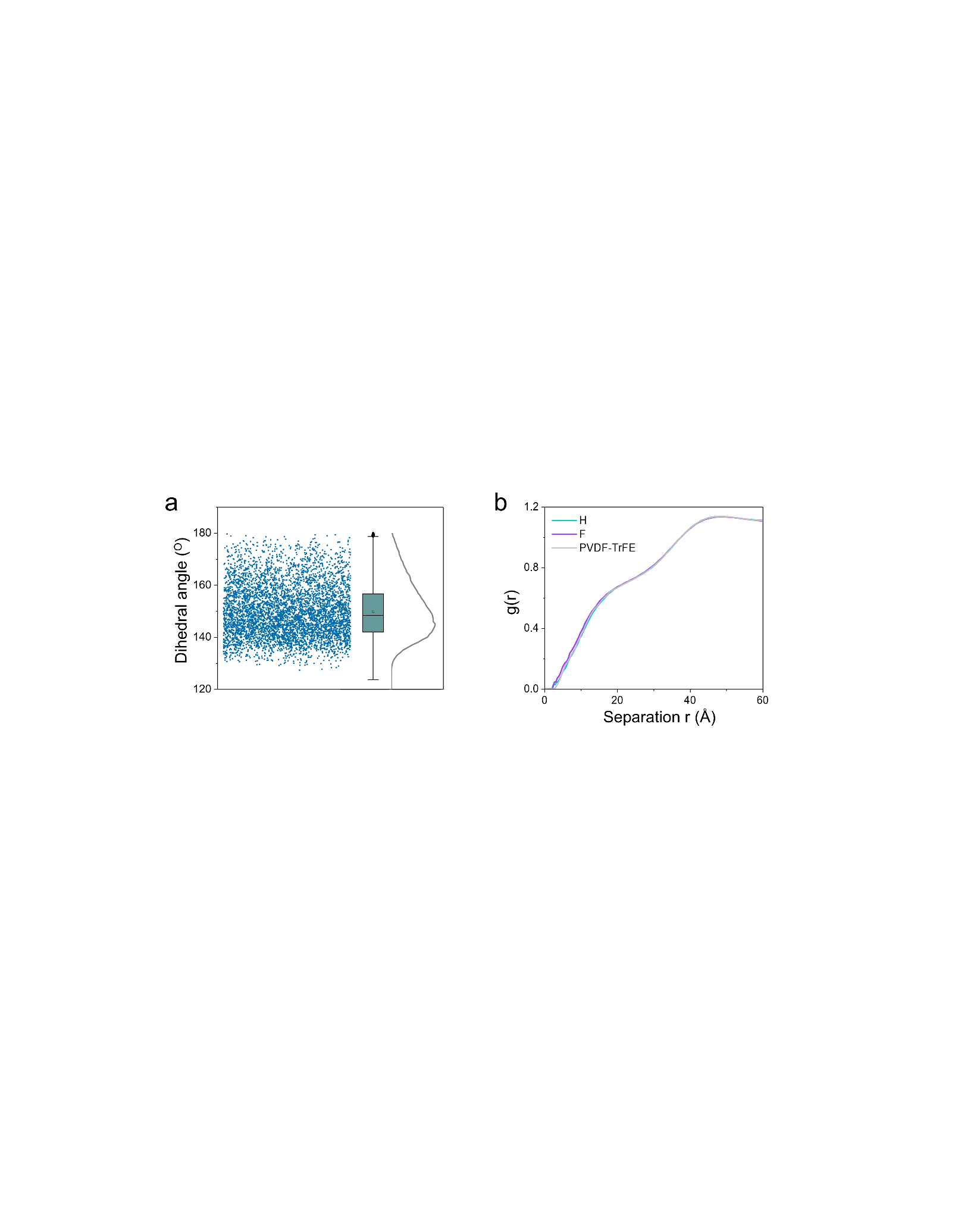
**

**Fig. S5** Analysis of the hydrogen bonding characteristics between the MXene and PVDF-TrFE molecular chains. **a** Statistics of hydrogen bond angles throughout the entire time period and the distribution analysis. **b** Radial distribution function of PVDF-TrFE molecular chains and H, F on it

**
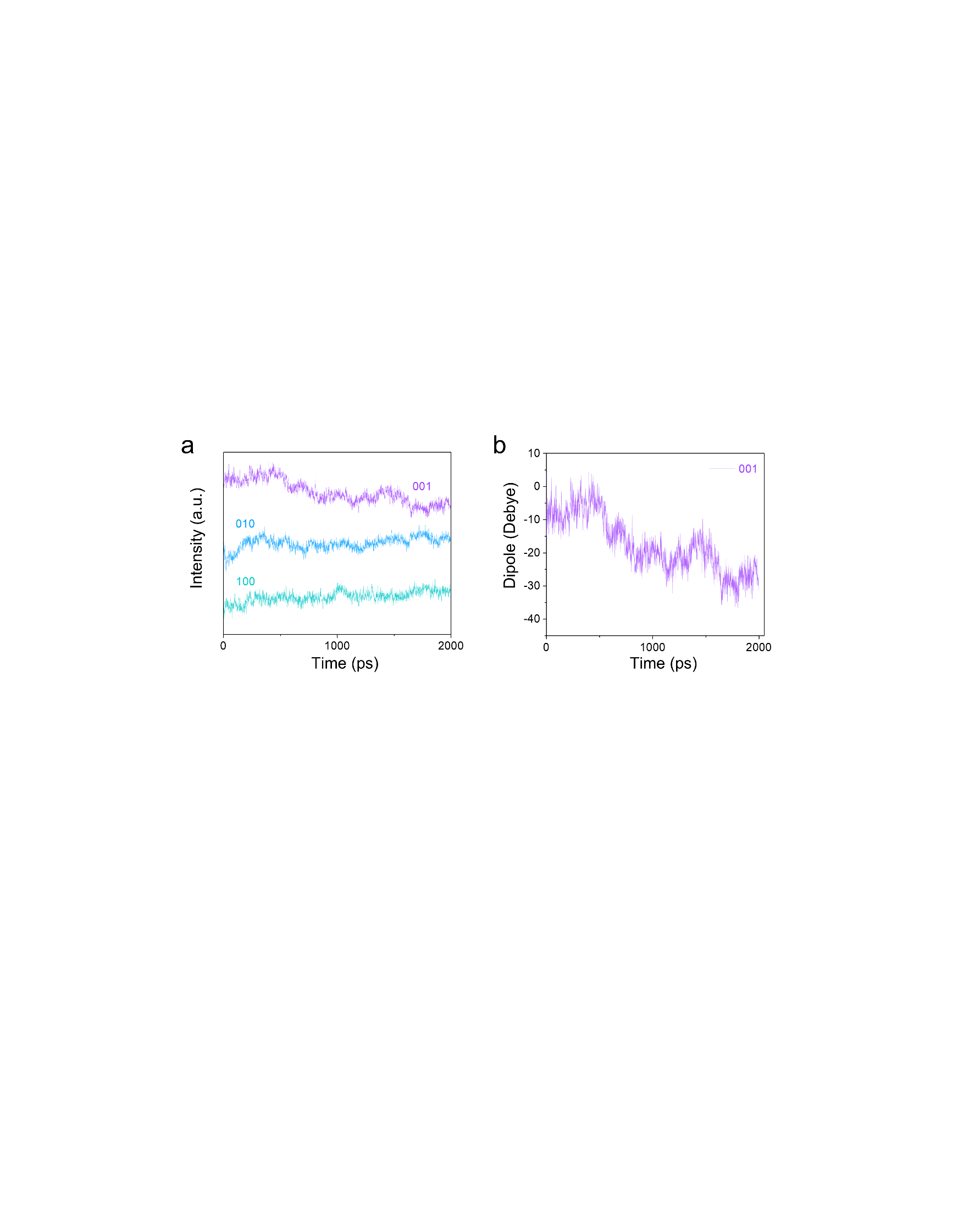
**

**Fig. S6** Dipole evolution analysis of the overall PVDF-TrFE molecular chains in [100], [010], [001] three directions. **a** Comparative dipole evolution in [100], [010], [001] three directions as a function of time. **b** Dipole intensity variation diagram in [001] direction

**
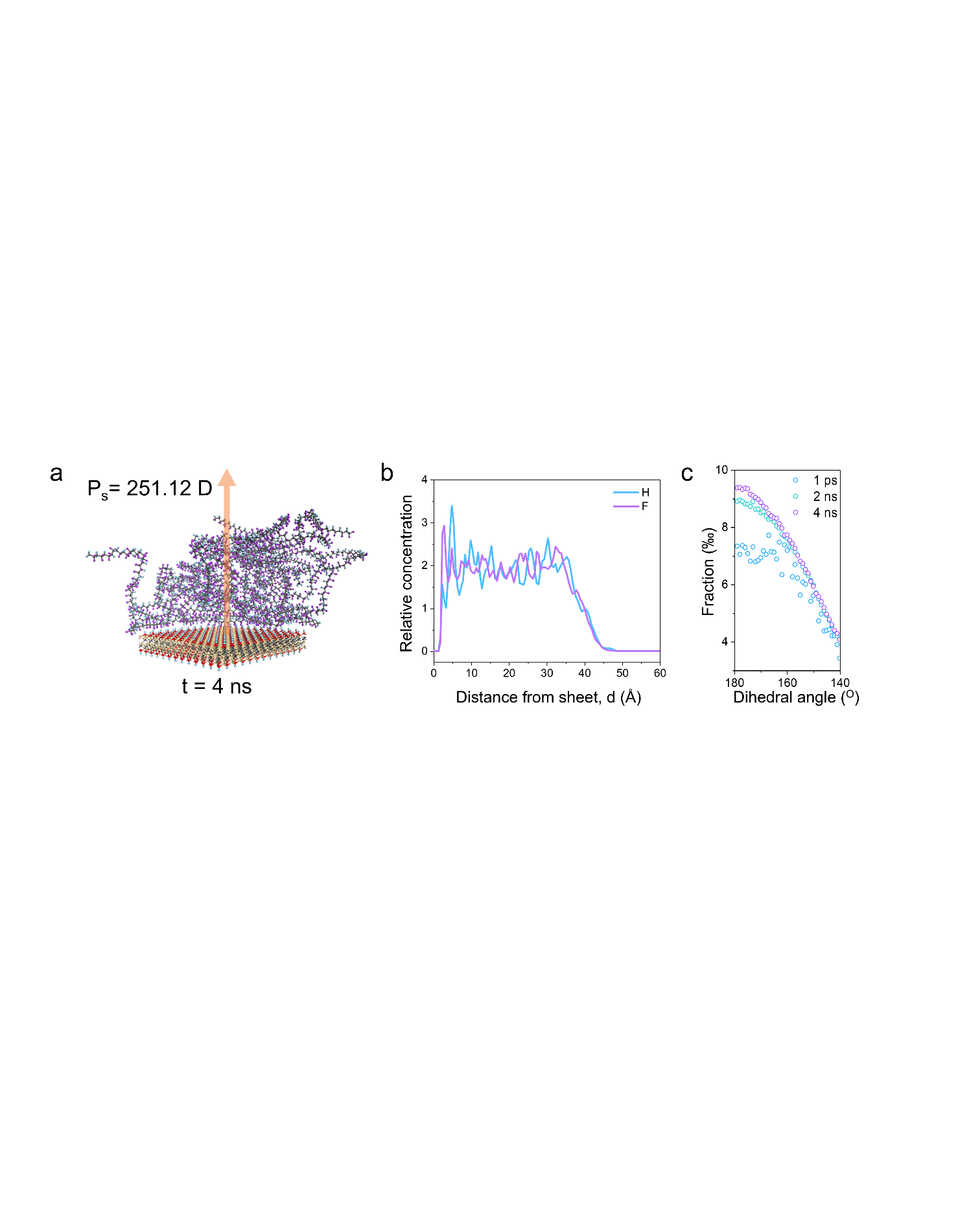
**

**Fig. S7** MD simulations of the overall system after balancing with an extra electric field for 2ns. **a** Final (t=4 ns) snapshot for MD simulations of the polarization of PVDF-TrFE at the interface. **b** Relative concentration distribution functions of H and F element on the surface of MXene nanosheets. **c** Comparative dihedral angles distribution of PVDF-TrFE chains near the 180^o^ (*all-trans*) at 1ps, averaged over 2 ns and 4 ns**
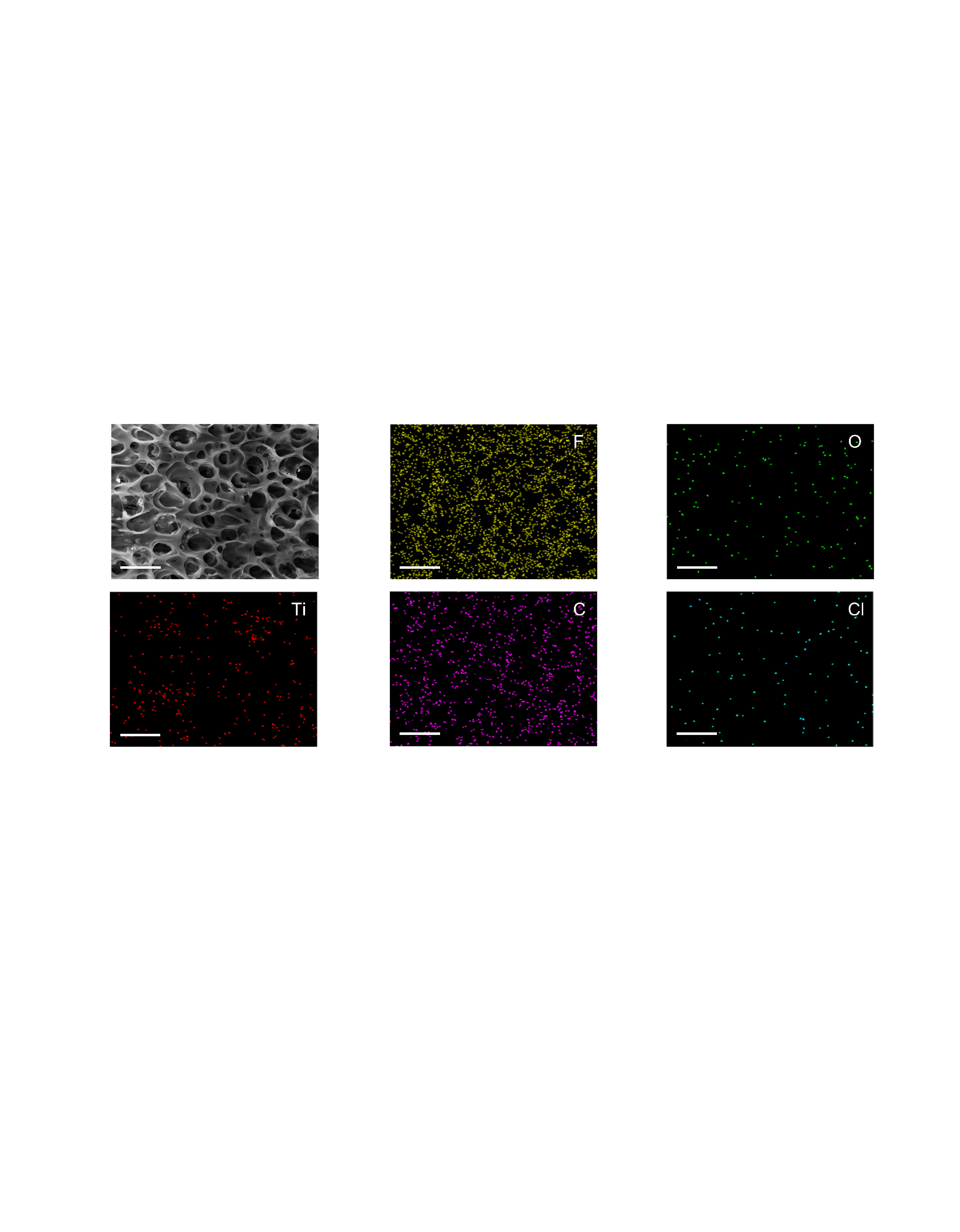
**

**Fig. S8** Characterization of morphology and elemental composition of porous MXene/PVDF-TrFE (MX-P) composite. SEM image and corresponding elements map of F, O, Ti, C, Cl derived from energy dispersive spectrometer


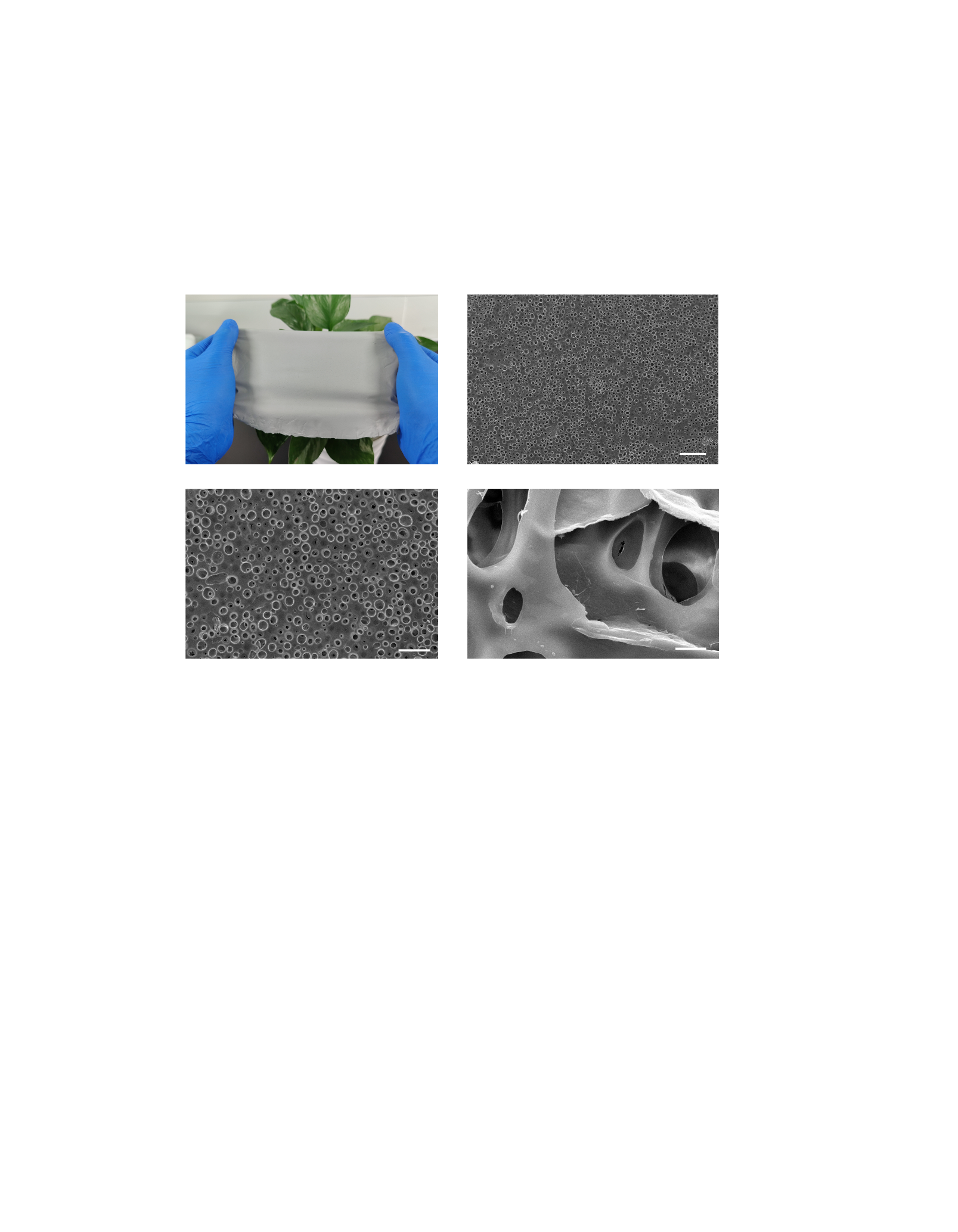


**Fig. S9** Photographic and SEM images of porous MX-P film. Scale bars are 100 μm, 50 μm and 5 μm, respectively. The images of porous MX-P film present the detail structure and potential for large-scale fabrication

**
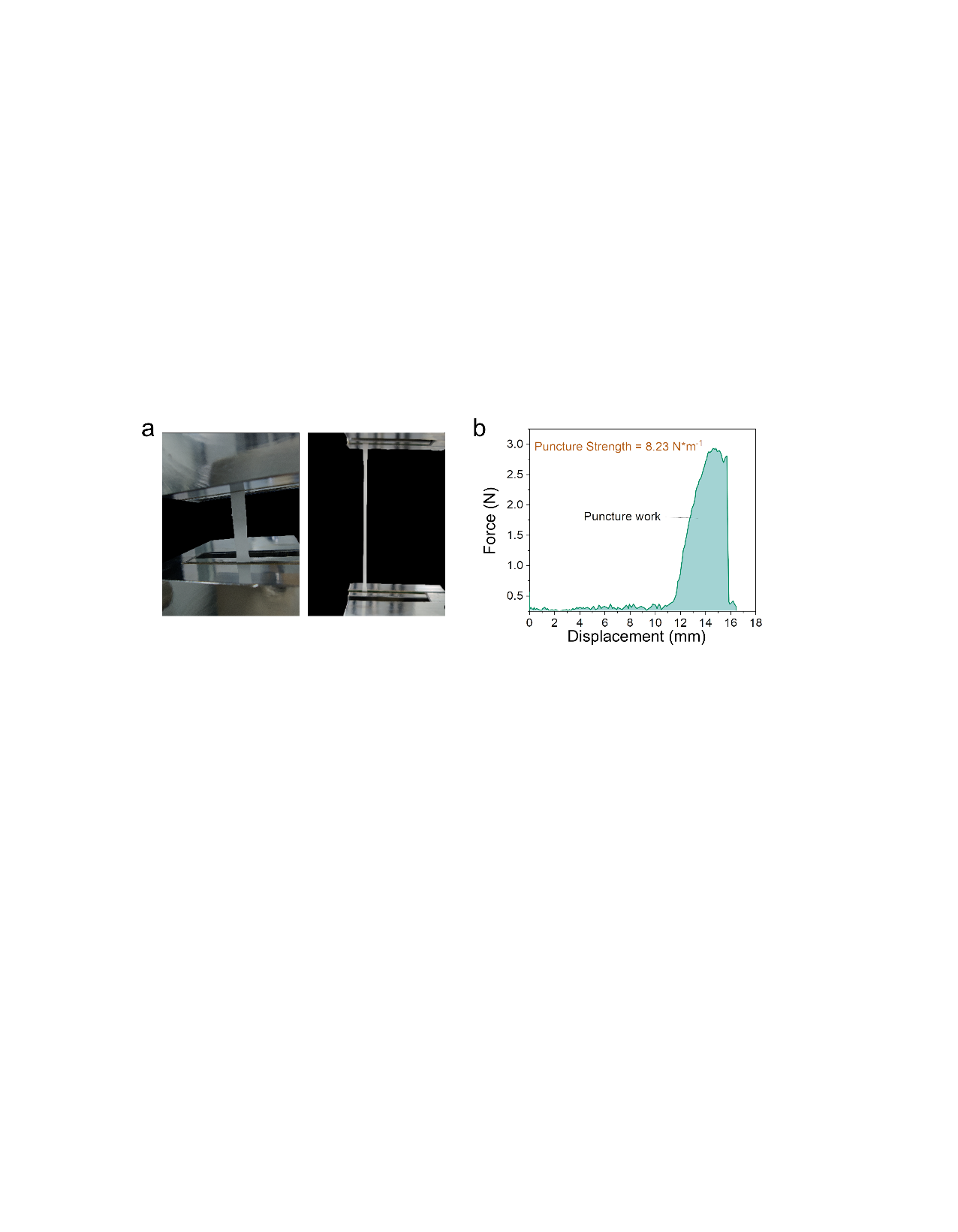
**

**Fig. S10** Mechanical characterization of porous MX-P. **a** Initial and post-strain digital images of porous MX-P under uniaxial tension. **b** Puncture strength measurement of porous MX-P

**
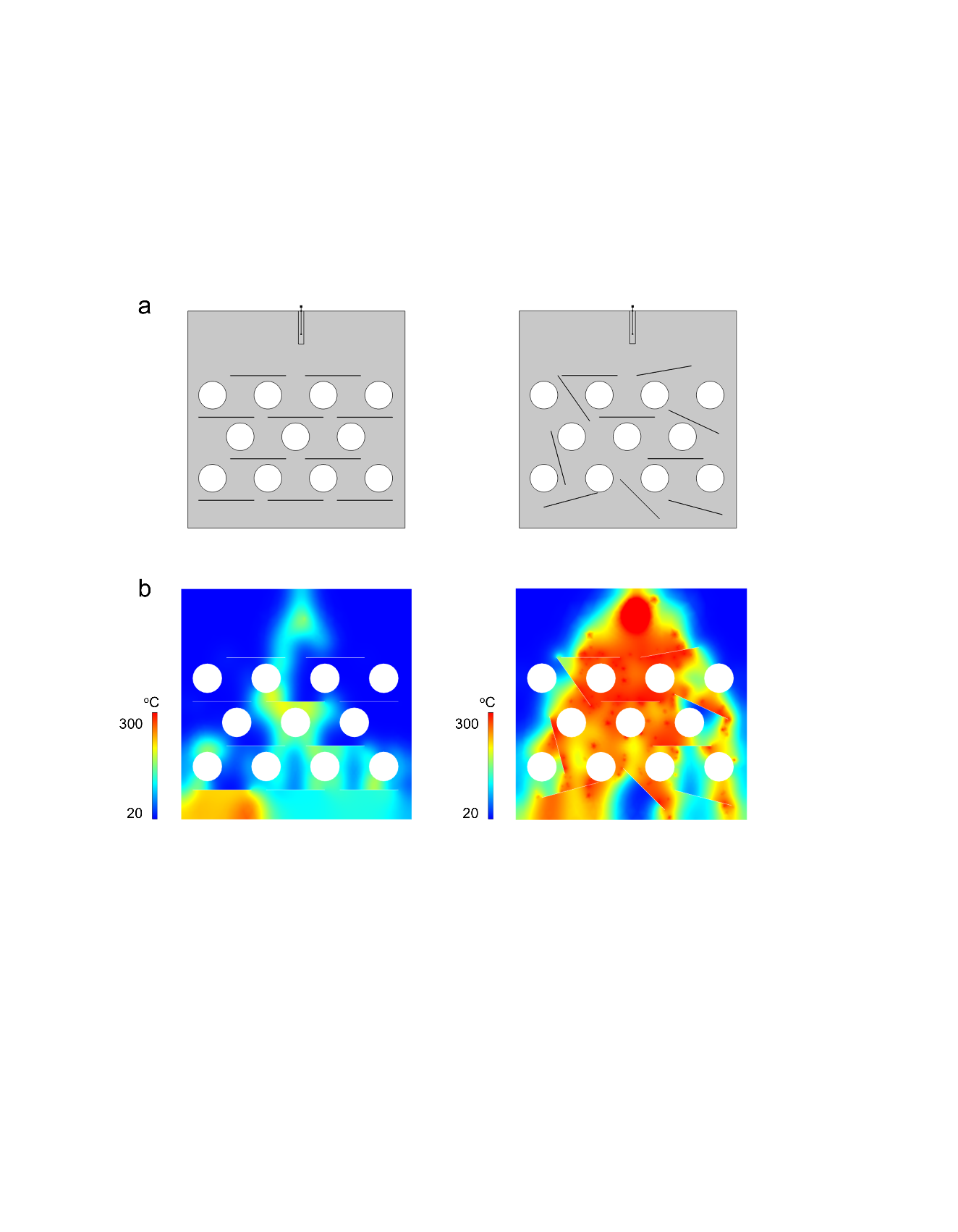
**

**Fig. S11** Model and temperature maps’ snapshots of dielectric breakdown simulations. **a** Models of composite with MXene oriented and random distribution. **b** The temperature maps’ snapshots of composite with MXene oriented and random distribution after breakdown

**
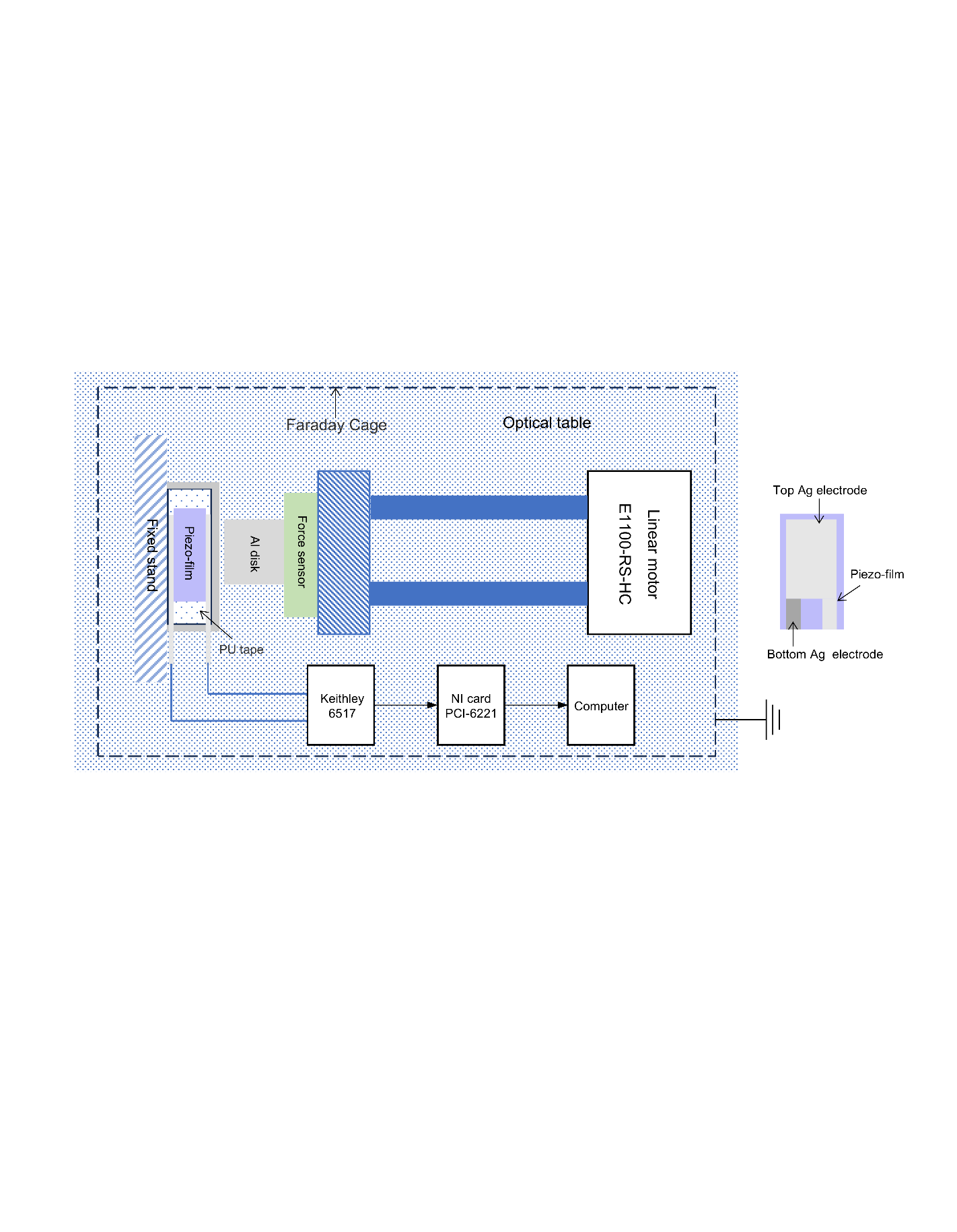
**

**Fig. S12** Schematic diagram of electric properties measurement setup. Steady dynamic force is supported by linear motor and optical table. The force values are recorded by force sensor in real time. Electric output was recorded by Keithley 6517, and the faraday cage shields the system from electrostatic charges and electromagnetic interference

**
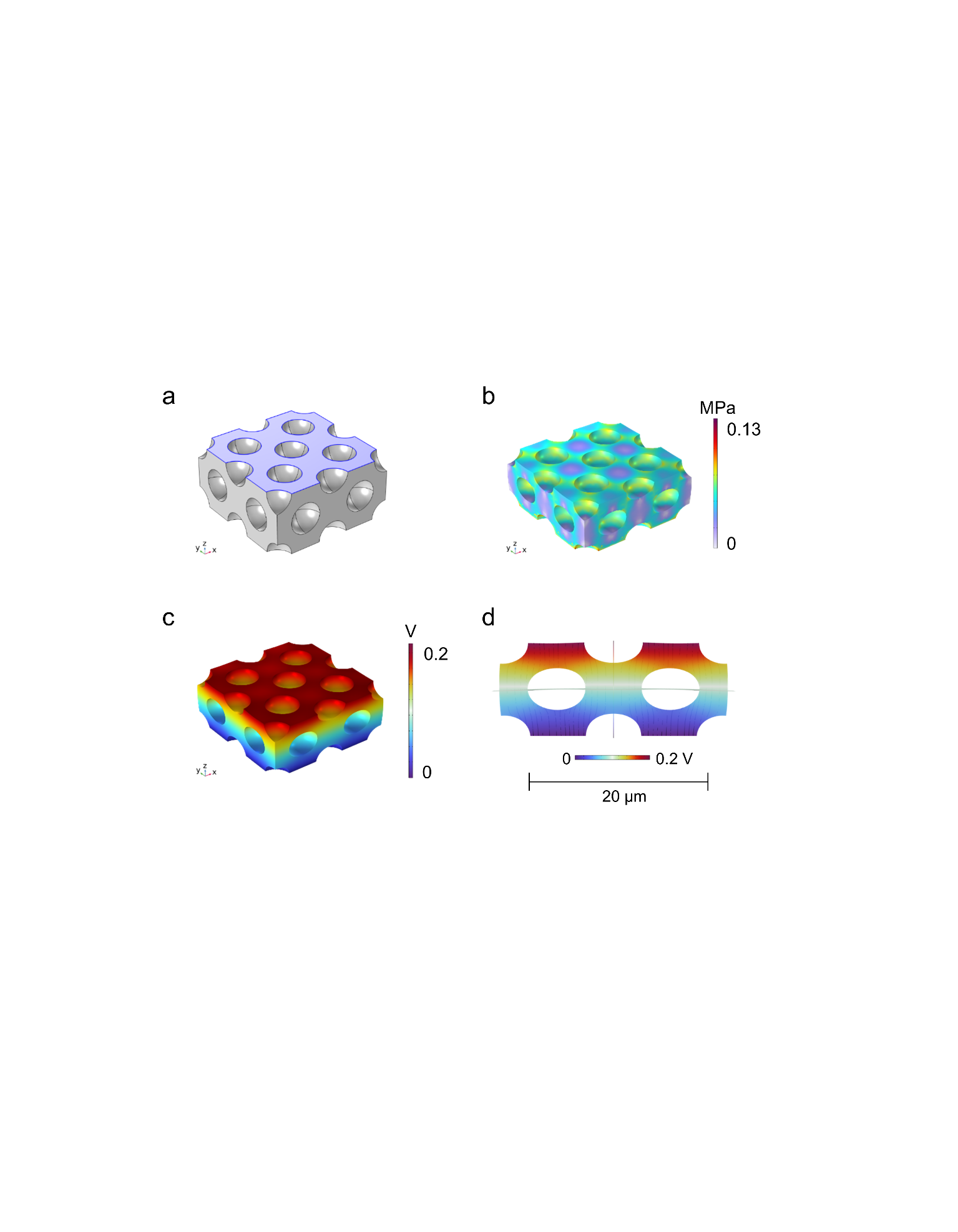
**

**Fig. S13** Pressure distribution simulation for porous PVDF-TrFE. **a** Model of the PVDF-TrFE with porous structure. **b** Pressure and **c** corresponding potential distribution of the porous PVDF-TrFE under pressure. **d** Vertical section of the potential distribution

**
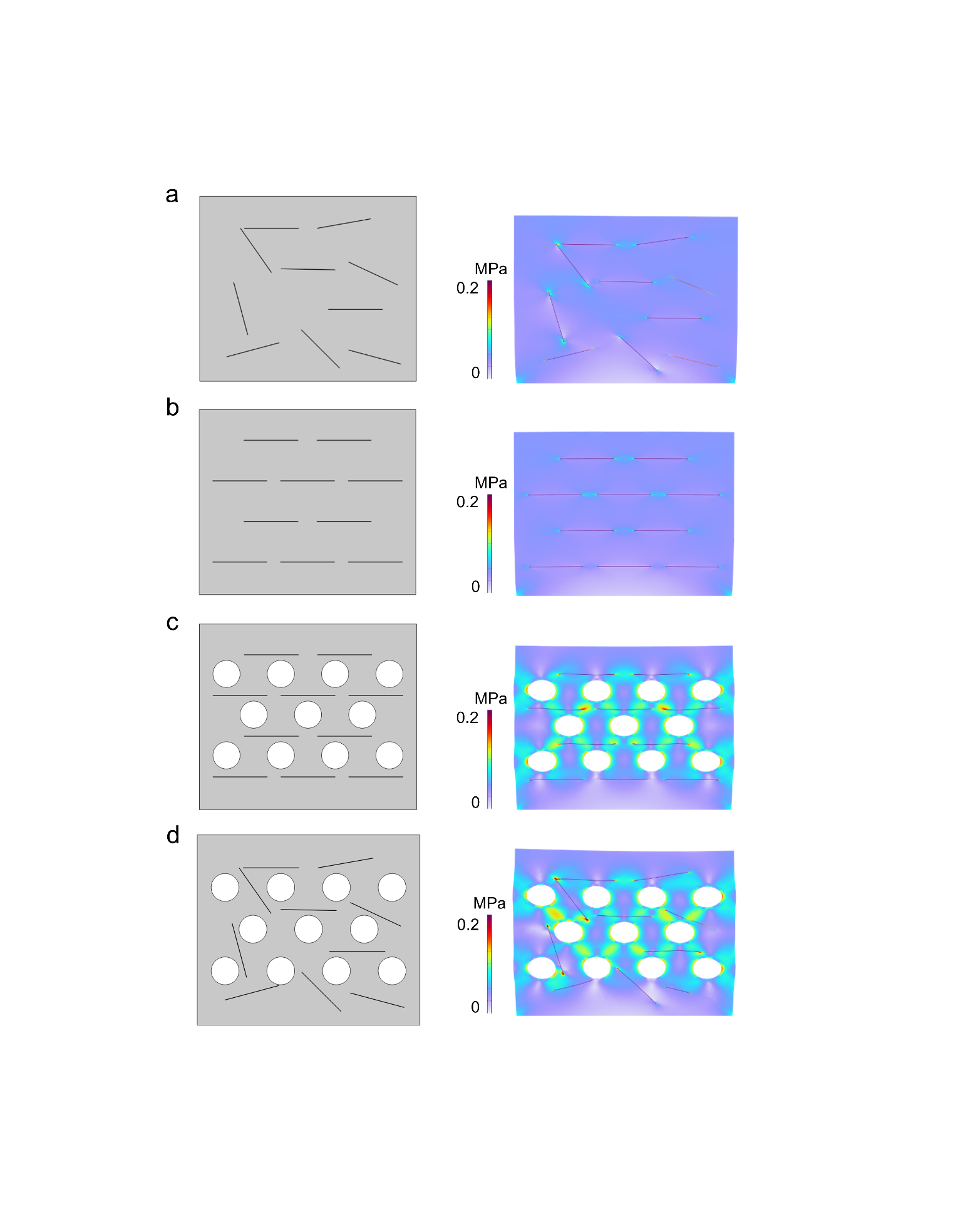
**

**Fig. S14** Models and pressure distribution simulation of MX-P with different structure. **a** Dense MX-P with MXene random distribution. **b** Dense MX-P with MXene oriented distribution. **c** Porous MX-P with MXene random distribution. **d** Porous MX-P with MXene oriented distribution

**
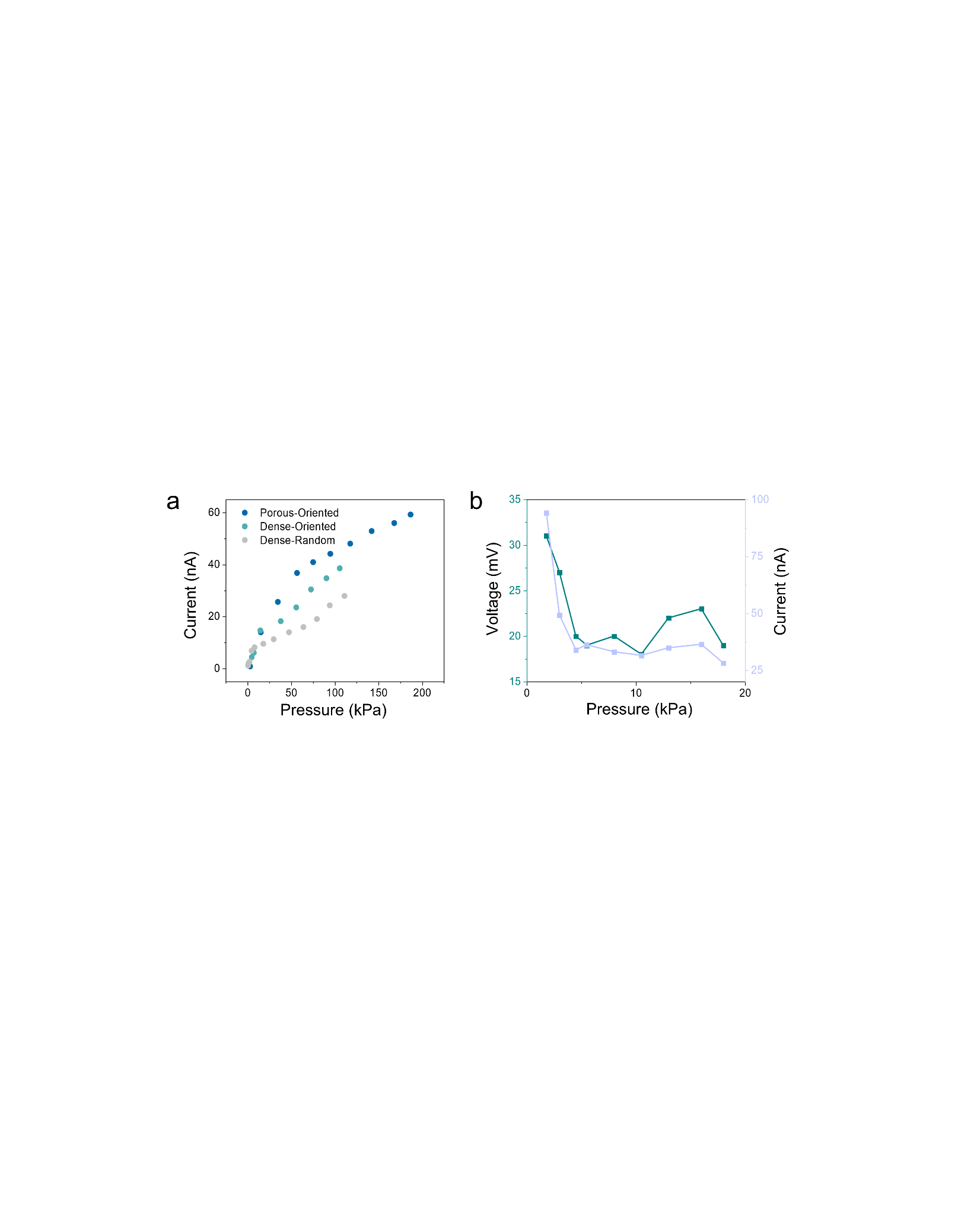
**

**Fig. S15** Electric output of different composite films. **a** Comparative electric output of porous MX-P with MXene oriented distribution (Porous-Oriented), dense MX-P with MXene oriented distribution (Dense-Oriented) and dense MX-P with MXene random distribution (Dense-Random). **b** Electric output of composite as a function of pressure at an excessive MXene concentration (7.5 wt%)

**
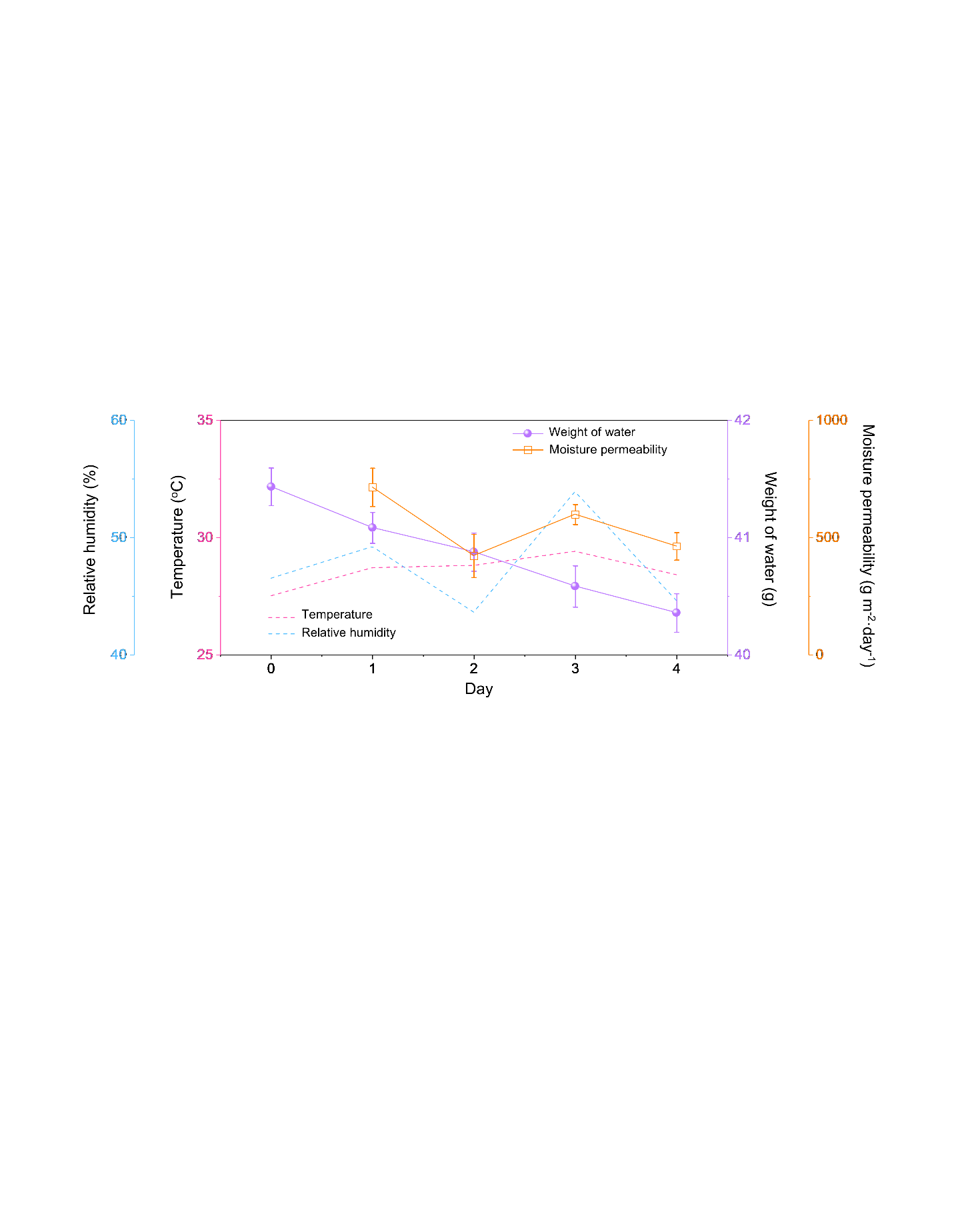
**

**Fig. S16** Moisture permeability measurement of composite film. The moisture permeability of porous composite film is about 500 g m^-2^·day^-1^, and it fluctuates with ambient humidity

**
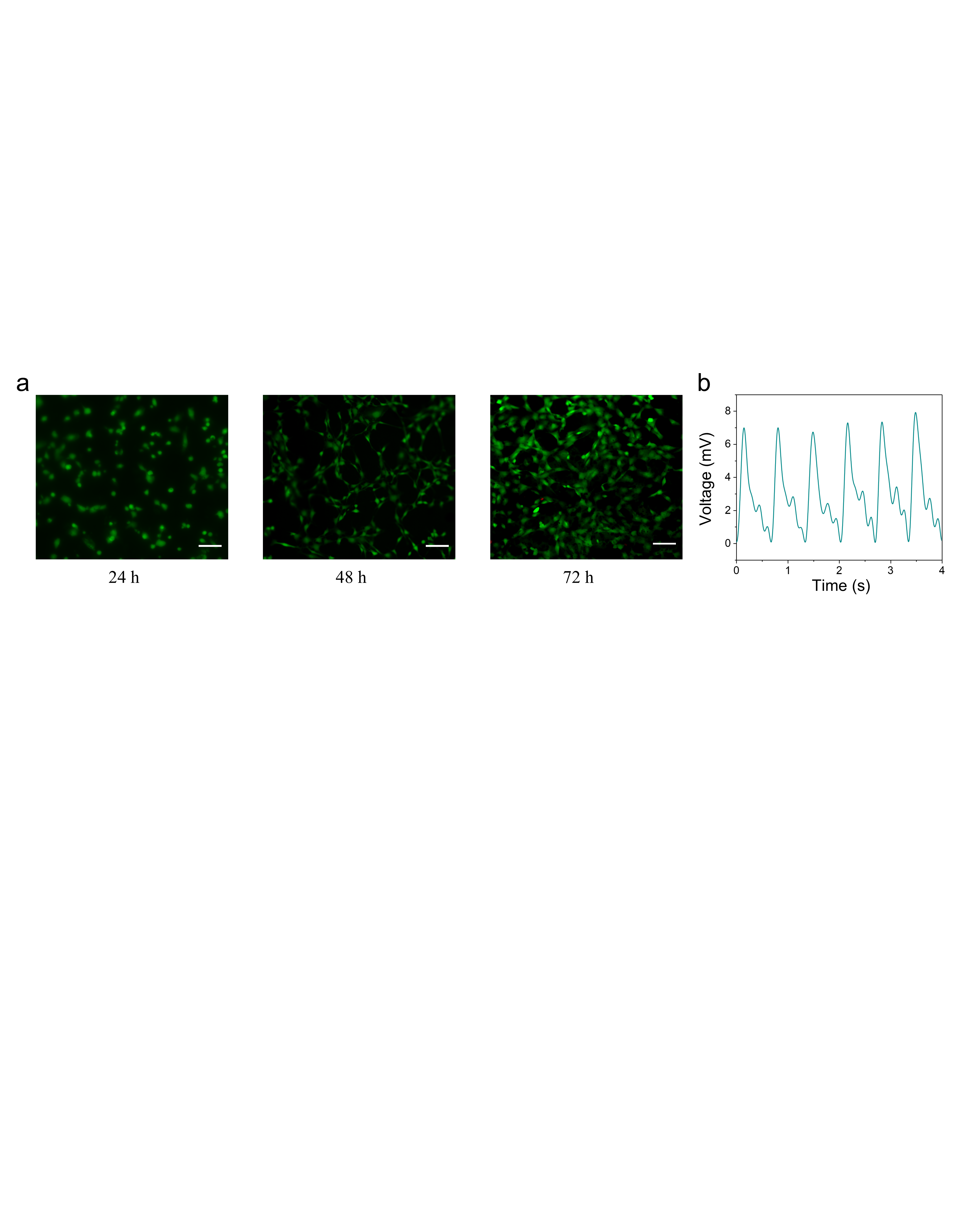
**

**Fig. S17** Cytotoxicity testing and radial artery pulse signal detection. **a** Live/dead staining results L929 fibroblasts cultured on porous MX-P after 72 h. Scale is 100 μm. Live cells are stained green and dead cells are stained red. **b** Radial artery pulse wave detected by the sensor attached to wrist

**
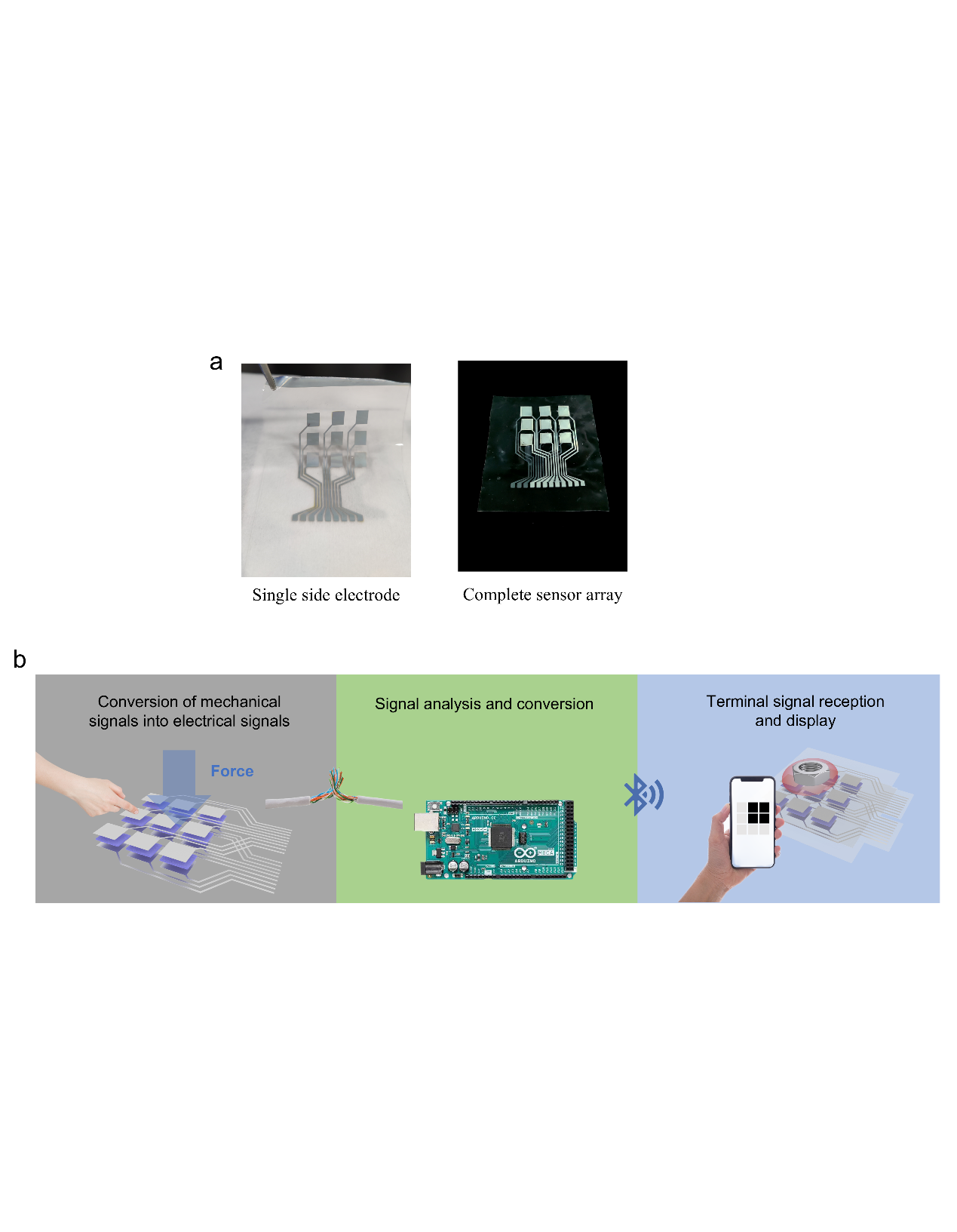
**

**Fig. S18** Display of the sensor array fabrication and schematic diagram of wireless pressure detection system. **a** Single side electrode on PDMS (left) and complete sensor array (right). **b** The wireless pressure detection system is composed of sensor array, SCM and mobile phone as termination

**
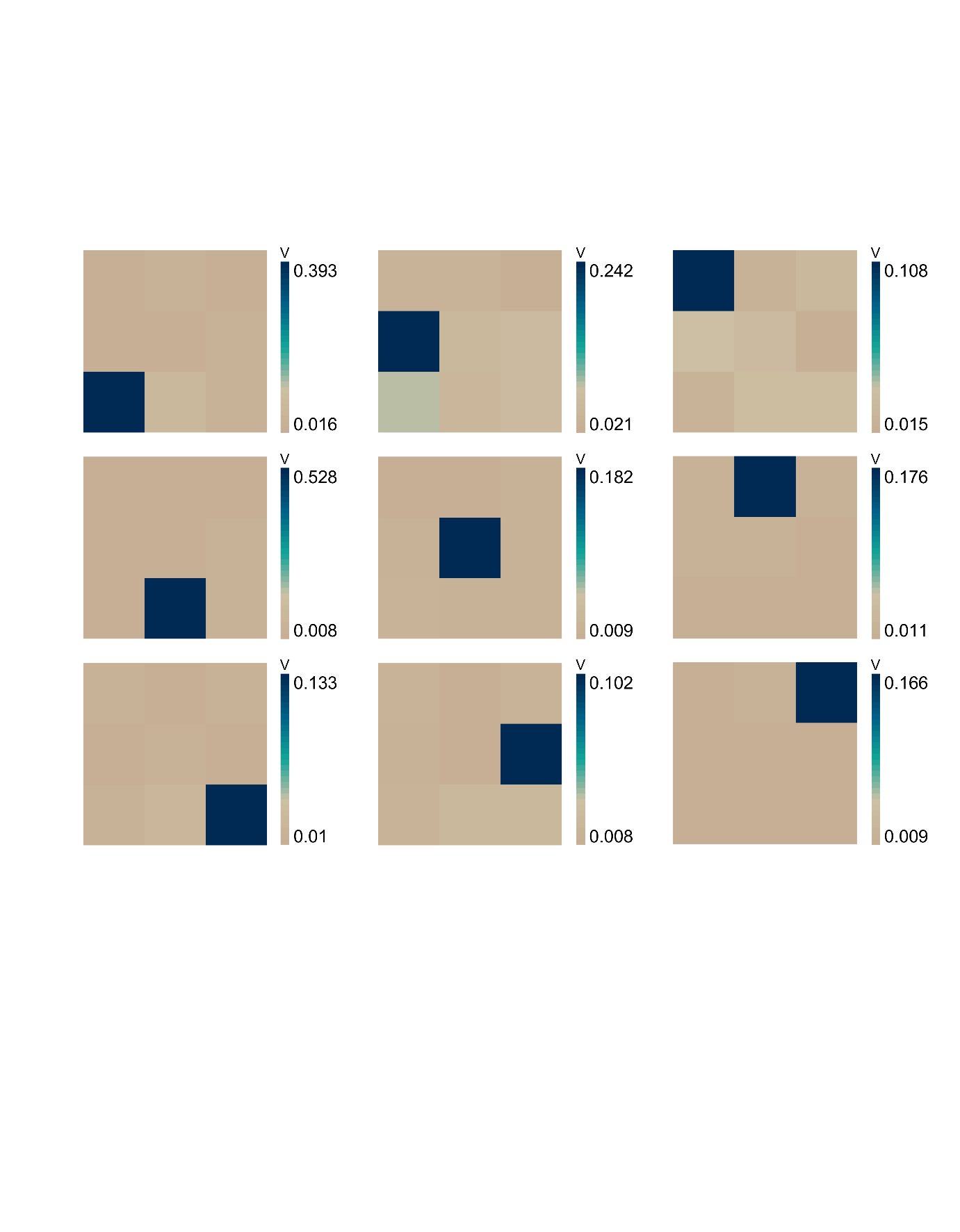
**

**Fig. S19** Single point test of sensor array. Each sensor in array can detect pressure independently with high signal-to-noise ratio

**Supplementary Tables**

**Table S1** Structural characteristics of porous MX-P composite

| Total intrusion volume: 2.2956 ml g^-1^ |
| --- |
| Total pore area: 7.737 m^2^ g^-1^ |
| Median pore diameter (volume): 1722.36 nm |
| Average pore diameter (4V/A): 1186.76 nm |
| Permeability: 2828.9235 mdarcy |
| Porosity: 76.00778% |
| Characteristic length: 78002.48 nm |
| Tortuosity factor: 1.37 |
| Calculated porosity: 76.0778% |

**Table S2** Comparison of piezoelectric performance between different materials

| Nanocomposite | Stability (cycles) | Sensitivity | Output current (nA) | References |
| --- | --- | --- | --- | --- |
| CNT/PVDF | 15000 | 0.056 V N^-1^ | 60 | [S1] |
| MXene/PVDF | ─ | 0.048 V N^-1^ | ─ | [S2] |
| ZnO/PVDF | 5000 | 3.12 V MPa^-1^ | 10 | [S3] |
| ZnO | ─ | 0.5 V MPa^-1^ | 7 | [S4] |
| PVDF-TrFE | 3000 | 4.56 V MPa^-1^ | ─ | [S5] |
| GaFeO_3_/PVDF | ─ | ─ | 4 | [S6] |
| hβ-PVDF | 10000 | ─ | 30 | [S7] |
| This work | 24000 | 6.39 V MPa^-1^ | 60 |  |

**Supplementary References**

1. W. Zeng, W. Deng, T. Yang, S. Wang, Y. Sun et al., Gradient CNT/PVDF piezoelectric composite with enhanced force-electric coupling for soccer training. Nano Res. **16**(8), 11312–11319 (2023). <https://doi.org/10.1007/s12274-023-5869-6>
2. Q. Zhao, L. Yang, Y. Ma, H. Huang, H. He et al., Highly sensitive, reliable and flexible pressure sensor based on piezoelectric PVDF hybrid film using MXene nanosheet reinforcement. J. Alloys Compd. **886**, 161069 (2021). <https://doi.org/10.1016/j.jallcom.2021.161069>
3. Z. Wang, Y. Sun, S. Wang, D. Xiong, G. Tian et al., Insight into piezoelectricity modulation mechanism of ZnO doped with Y ions. J. Mater. Chem. A **12**(21), 12435–12442 (2024). <https://doi.org/10.1039/D4TA01317D>
4. G. Tian, D. Xiong, Y. Su, T. Yang, Y. Gao et al., Understanding the potential screening effect through the discretely structured ZnO nanorods piezo array. Nano Lett. **20**(6), 4270–4277 (2020). <https://doi.org/10.1021/acs.nanolett.0c00793>
5. G. Tian, L. Tang, J. Zhang, S. Wang, Y. Sun et al., Ultrathin epidermal P(VDF-TrFE) piezoelectric film for wearable electronics. ACS Appl. Electron. Mater. **5**(3), 1730–1737 (2023). <https://doi.org/10.1021/acsaelm.2c01760>
6. M. Mishra, A. Roy, S. Dash, S. Mukherjee, Flexible nano-GFO/PVDF piezoelectric-polymer nano-composite films for mechanical energy harvesting. IOP Conf. Ser. Mater. Sci. Eng. **338**(1), 012026 (2018). <https://doi.org/10.1088/1757-899X/338/1/012026>
7. L. Jin, S. Ma, W. Deng, C. Yan, T. Yang et al., Polarization-free high-crystallization β-PVDF piezoelectric nanogenerator toward self-powered 3D acceleration sensor. Nano Energy **50**, 632–638 (2018). <https://doi.org/10.1016/j.nanoen.2018.05.068>
